# The Global Navigation Satellite Systems Reflectometry (GNSS-R) Microwave Interferometric Reflectometer: Hardware, Calibration, and Validation Experiments

**DOI:** 10.3390/s19051019

**Published:** 2019-02-27

**Authors:** Raul Onrubia, Daniel Pascual, Jorge Querol, Hyuk Park, Adriano Camps

**Affiliations:** 1 Remote Sensing Lab (RSLab), Department of Signal Theory and Communications (TSC), Universitat Politècnica de Catalunya—BarcelonaTech (UPC), Jordi Girona 1-3, Campus Nord, D4 Building, 08034 Barcelona, Spain; jorge.querol@tsc.upc.edu (J.Q.); park.hyuk@tsc.upc.edu (H.P.); 2 Institut d’Estudis Espacials de Catalunya (IEEC/UPC), 08034 Barcelona, Spain; 3 Physiscs Department, School of Telecommunications and Aerospace Engineering (EETAC), Universitat Politècnica de Catalunya—BarcelonaTech (UPC), 08860 Castelldefels, Spain

**Keywords:** GNSS, Reflectometry, remote sensing, antenna array, beamforming, beam steering, calibration

## Abstract

This manuscript describes the Microwave Interferometric Reflectometer (MIR) instrument, a multi-beam dual-band GNSS-Reflectometer with beam-steering capabilities built to assess the performance of a PAssive Reflectrometry and Interferometry System—In Orbit Demonstrator (PARIS-IoD) like instrument and to compare the performance of different GNSS-R techniques and signals. The instrument is capable of tracking up to 4 different GNSS satellites, two at L1/E1 band, and two at L5/E5 band. The calibration procedure of the up- and down-looking arrays is presented, the calibration performance is evaluated, and the results of the validation experiments carried out before the field experiments are shown in this paper.

## 1. Introduction

When Global Navigation Satellite Systems—Reflectometry (GNSS-R) was first proposed in 1988 for multistatic scatterometric applications [1], the Global Positioning System (GPS) was the only one available and with a limited number of broadcasted signals. At that time, the only publicly available signal was the L1 C/A, which has a narrow bandwidth (2 MHz), and therefore limited ranging accuracy, while the private signals such as the precise code P(Y), and the military signal M have a wider spectrum (≈20–30 MHz). In order to take advantage of the wide spectrum signals, the so called interferometric technique (iGNSS-R) was proposed in 1993 [2]. It consists of the correlation between the reflected signals on the Earth’s surface and the signal received directly from the same transmitting satellite. In this way, it is not necessary to know the transmitted codes, and it is possible to take advantage of the wider signals. However, since both signals are received with a poor Signal-to-Noise Ratio (SNR), high directive antennas are needed. Besides, the correlation of the two received signals is sensitive to be affected by Radio Frequency Interference (RFI) [3], and by satellite cross-talk [4]. Later on, the nowadays called conventional technique (cGNSS-R) was proposed in 1996 [5]. It consists on the cross-correlation between the reflected signals with a locally generated clean replica of the transmitted code. In this way, the achieved SNR is higher and the effect of other satellites and RFI is mitigated, but only the narrow-band open signals can be used.

Nowadays, more GNSS systems have been deployed and wider signals are being broadcasted, such as the public Galileo E5 signal, with more than 40 MHz bandwidth [6]. While a performance comparison of these signals has been studied with simulated data [7], they have never been compared with real data in similar conditions. For this reason, during the past years a new airborne instrument has been developed at Universitat Politècnica de Catalunya: the Microwave Interferometric Reflectometer (MIR).

This manuscript describes the MIR instrument, the calibration procedure, and shows the validation results of the experiments that were conducted to prove its performance.

## 2. Instrument Overview

**Figure 1:**
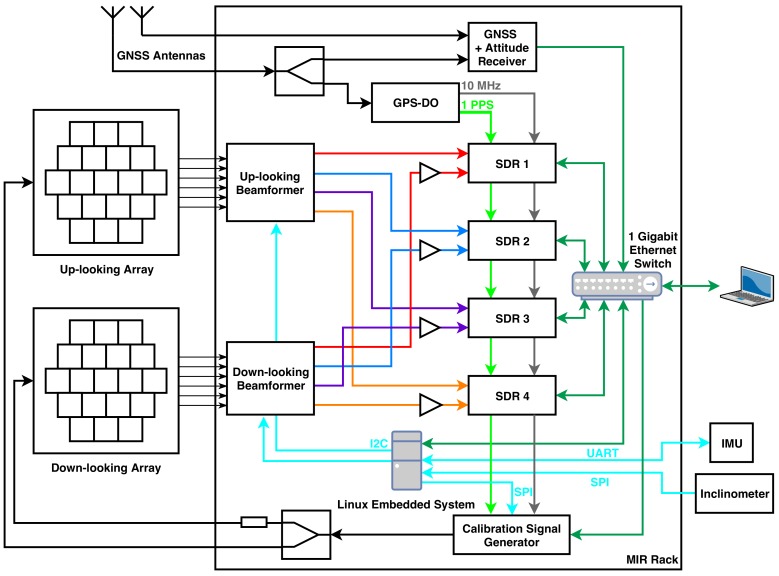
Block diagram of the MIR instrument. The red, blue, orange, and purple lines correspond to the radio frequency (RF) signals of each of the four beams from both the up- and the down-looking array. The light green is a 1 Pulse Per Second (PPS) signal. The grey line is a 10 MHz reference signal. The cyan lines correspond to serial protocols of communication, such as Inter-Integrated Circuit (I2C), Serial Peripheral Interface (SPI) or Universal Asynchronous Receiver-Transmitter (UART). The dark green lines are Ethernet lines. The black lines are other radio frequency (RF) signals, such as the calibration signals, or the GNSS signals for the GNSS receiver. SDR stands for Software Defined Radio. IMU stands for Inertial Measurement Unit. GPS-DO stands for GPS—Disciplined Oscillator.

The MIR instrument is a GNSS reflectometer that has two steerable arrays. The up-looking one points directly to the GNSS satellites, while the down-looking one points where the signals from these satellites are reflected on the Earth’s surface. Each of these arrays is connected to an analog beamformer that creates two beams at L1/E1 (1575.42 MHz) and two beams at L5/E5a (1176.45 MHz), therefore the instrument can track up to 4 different satellites, and their corresponding specular reflection points. The signals received from a satellite by the up-looking array are sampled synchronously by pairs with the signal from the same satellite received by the down-looking array after reflecting on the Earth’s surface. The signals coming from the down-looking array are amplified prior to be sampled in order to compensate the losses caused by scattering from various landscapes, including rough and vegetated ground. The data is sampled using SDRs and then is stored for post-processing. The instrument is controlled by a Linux embedded computer that selects the best available satellites and points the instrument beams towards them, and to the expected specular reflection points taking into account the position of the satellites and the position and attitude of the plane. Last, the instrument has a calibration system that generates PseudoRandom Noise (PRN) codes to calibrate the instrument in amplitude and phase. Figure 1 shows the instrument block diagram. The next subsections explain all blocks in detail.

### 2.1. Antenna Array and RF Front-Ends

**Figure 2:**
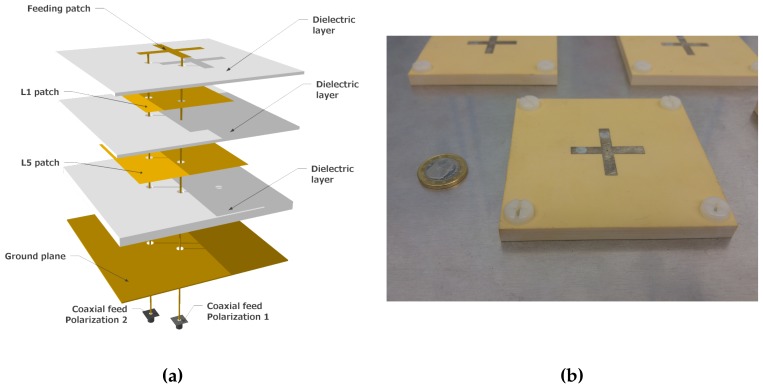
3D model of the antenna elements (**a**), and photography of one of the manufactured antennas (**b**). The antenna measures 95 × 95 × 8 mm.

MIR has two antenna arrays, an up-looking Right Hand Circular Polarized (RHCP), and a down-looking Left Hand Circular Polarized (LHCP) one. Each array has 19 dual-band patch antennas. The antenna elements are dual-band patch antennas of about 95 * 95 * 8 mm specifically designed for the instrument [8]. The antennas (Figure 2) are made of two square patches stacked between dielectric substrates (Rogers 4003c) that resonate at the L1/E1 central frequency (1575.42 GHz), and at the E5 central frequency (1191.795 GHz), respectively. The antenna is fed by capacitive coupling using a cross-shaped patch on the top of the antenna, which is itself electrically fed by two probes. The cross-shape of the feeding patch was found to be useful to increase the bandwidth of the antenna, but at the expense of reducing the axial ratio of the antenna [8]. A thick dielectric layer between the L5 patch and the ground plane was used to increase the bandwidth. The feeding patch dimensions and the position of the feeding coaxial cables/connectors along the antenna axes were optimized to maximize the bandwidth and the matching of the antenna at the aforementioned frequency bands.

**Figure 3:**
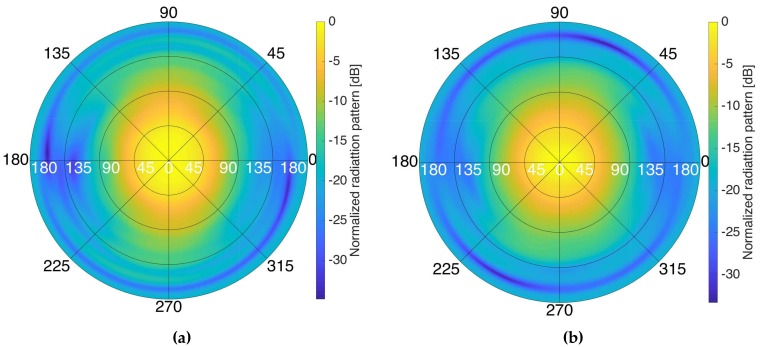
Measured antenna radiation pattern of one of the manufactured antennas at L1/E1 (**a**), and at L5/E5 (**b**).

**Figure 4:**
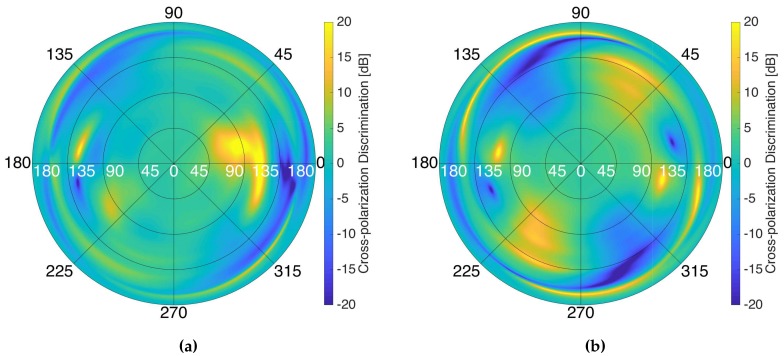
Measured cross-polarization discrimination patterns at L1/E1 (**a**), and L5/E5a (**b**) frequency bands. The colorscale was limited to ±20 dB to ease the viewing.

Four manufactured elements were measured and they showed an average bandwidth at −10 dB of ≈20 MHz at L1/E1, and ≈34 MHz at L5/E5a. The antenna radiation pattern of one of the antenna elements at the different frequency bands was measured at both frequency bands (Figure 3) in the Universitat Politècnica de Catalunya (UPC) AntennaLab anechoic chamber, and also the cross-polarization pattern (see Figure 4). The antenna directivities in each band are shown in Table 1. The efficiency was measured using calibrated reference antennas.

**Figure 5:**
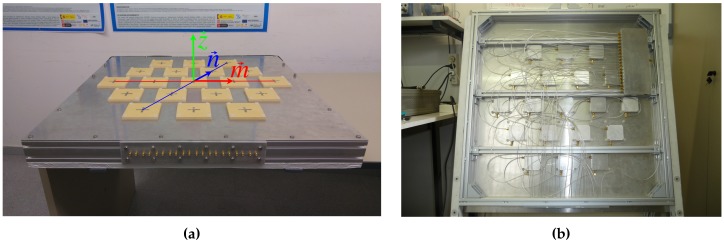
Top view of the up-looking array (**a**), and back view (uncovered) of the down-looking array (**b**). The up-looking array image includes the baseline used to define each antenna element by a $\left\{m_{i},n_{i}\right\}$ pair of values. The azimuthal pointing angle φ is defined anti-clockwise from vector $\vec{m}$ to $\vec{n}$. The off-boresight pointing angle θ is defined from vector $\vec{z}$ to the antenna array ground plane.

The antennas are separated 0.75$\lambda_{L1}$ and 0.56$\lambda_{L5}$ over a regular hexagonal grid. The down-looking array is mounted in a squared ground plane of 860 mm per side that weights 25 kg approximately, and the up-looking one in a ground plane of 800 mm per side that weights 18 kg approximately (Figure 5). The arrays have a 19-ports power splitter attached to the structure. These devices split the calibration signal and inject the resulting signals to each Radio Frequency (RF) Front-end. The directivity of both arrays was measured in the anechoic chamber as explained later in Section 4.1 and is shown in Table 2.

The RF Front-ends are attached directly behind the antennas. A 90${}^{\circ }$ hybrid combines the two linear polarizations to generate a circular polarization: RHCP for the zenith looking antenna, and LHCP for the nadir looking one. The outgoing signal is then connected to one of the inputs of an RF switch. The other input is used to inject a calibration signal. The switch is always commuted to the antenna signal, behaving thus as a directional coupler. The combined signals are amplified with a Low Noise Amplifier (LNA) (G ≈ 15 dB, NF ≈ 0.8 dB), high-pass filtered ($F_{c}=880$ MHz) to attenuate broadcasted Frequency Modulation (FM) signals (80–100 MHz) and Global System for Mobile communications (GSM) signals (800–900 MHz), amplified again (G ≈ 22 dB, NF ≈ 2.1 dB), and low-pass filtered ($F_{c}=1825$ MHz) to attenuate broadcasted GSM signals (1700–1900 MHz) and Wi-Fi signals (2.4 GHz), and to avoid undesired high-frequency oscillations. Wi-Fi signals might not affect during flight operations, but are filtered to avoid any interference in laboratory tests.

### 2.2. Beamformer

**Figure 6:**
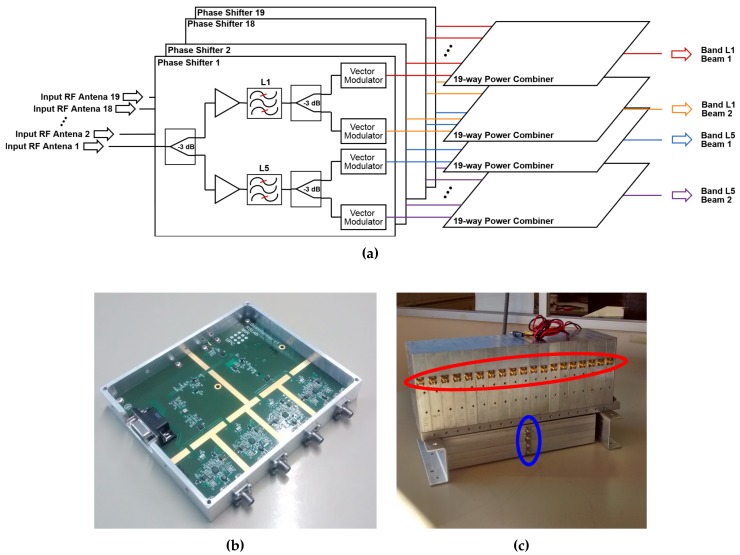
Block diagram of one of the beamformers (**a**), close view of one of the phase shifters manufactured (**b**), and front picture of one of the assembled beamformers (**c**). Note in the assembled beamformer 19 inputs (red), and the 4 outputs (blue).

Since digital beamforming would require an enormous amount of resources to be implemented (2 bands × 2 arrays × 19 antennas = 76 data streams to be sampled simultaneously and stored), two analog beamformers were implemented. Each beamformer block (Figure 6) consists of 19 boards that generate 4 different analog steerable beams for each array. Each board is connected to an RF Front-end. First, it divides the incoming signal into two branches, then they are amplified (G = 17 dB), and then they are band-pass filtered at the L1, or the L5 bands using Surface Acoustic Wave (SAW) filters. The L1 filter is centered at 1583 MHz, and has a bandwidth at −3 dB of 58 MHz. The L5 filter is centered at 1178.12 MHz and has a bandwidth at −3 dB of 55 MHz. Each of these branches is split again to generate two different beams for each band. The amplitude and phase of these signals is then modified to steer the beams electronically to the desired direction. Last, the 19 signals of each beam are combined with a 19-port power combiner, obtaining thus 4 RF lines, one for each particular band and beam. There is one of these beamformer blocks for each array.

After the scattering on the Earth’s surface, the reflected signal power in an airborne case is expected to be 0–30 dB lower than the direct one [9]. In order to compensate this expected power loss, two RF amplifiers with a total gain of ≈35 dB were placed after the beamformer.

**Figure 7:**
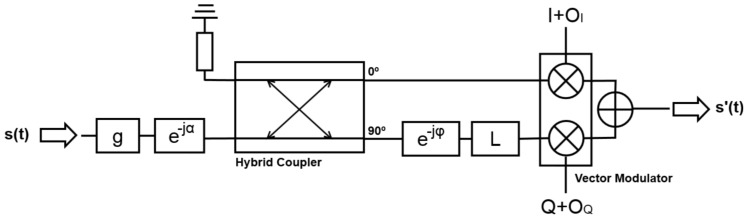
Used model for a whole RF chain.

The beam-forming was implemented with ADL5390 Vector Modulators [10], which are used as variable amplifiers and phase shifters. This device (Figure 7) amplifies or attenuates individually the *I* and *Q* components of the signal according to the voltage applied to the gain control pins. In this way, the magnitude and phase of the input is changed. Without any unbalance introduced by the vector modulator or the hybrid coupler, the vector modulator and the later combiner, the output signal is

(1) $$s^{\prime }\left(I,Q,t\right)=s\left(t\right)\cdot \left(I+j\cdot Q\right)\cdot g\cdot e^{j\omega_{c}t-j\alpha }=s\left(t\right)\cdot g\cdot \sqrt{{\left|I\right|}^{2}+{\left|Q\right|}^{2}}\cdot e^{j\cdot atan2(Q,I)}\cdot e^{j\omega_{c}t-j\alpha },$$

where *I* and *Q* are the voltage gains in the *I* and *Q* channels, respectively (note that they can be negative), s(t) is the input signal, $s^{\prime }\left(t\right)$ is the output signal, *g* and α are the gain and phase in the whole RF chain (the phase in the *Q* branch is π/2 radians larger), $\omega_{c}$ is the carrier angular frequency, and atan2 is the 2-argument arctangent function.

If the unbalances are taken into account, the output signal in the frequency domain becomes

(2) $$\begin{array}{cc} s^{\prime }\left(I,Q,t\right) & =\left(\left(I+O_{I}\right)\cdot s\left(t\right)+j\cdot L\cdot \left(Q+O_{Q}\right)\cdot e^{-j\varphi }\cdot s\left(t\right)\right)\cdot g\cdot e^{j\omega_{c}t-j\alpha }, \end{array}$$

where *L* and φ are the amplitude and phase unbalance introduced by the hybrid coupler, the vector modulator, and the power combiner, and $O_{I}$ and $O_{Q}$ are the gain offsets in the *I* and *Q* channels, respectively. To simplify, the *I* and *Q* gains and the gain offsets will be written in vector notation as

(3) $$\begin{array}{cc} k_{I} & =g\cdot e^{-j\alpha }\\ k_{Q} & =g\cdot L\cdot e^{-j\varphi }\cdot e^{-j\alpha }\\ O & =g\cdot \left(O_{I}+j\cdot O_{Q}\cdot L\cdot e^{-j\varphi }\right)\cdot e^{-j\alpha }. \end{array}$$

Then, (2) becomes

(4) $$\begin{array}{cc} s^{\prime }\left(I,Q,t\right) & =s\left(t\right)\cdot \left(I\cdot k_{I}+j\cdot Q\cdot k_{Q}+O\right)\cdot e^{j\omega_{c}t}=s\left(t\right)\cdot H_{VM}\left(I,Q\right)\cdot e^{j\omega_{c}t}. \end{array}$$

**Figure 8:**
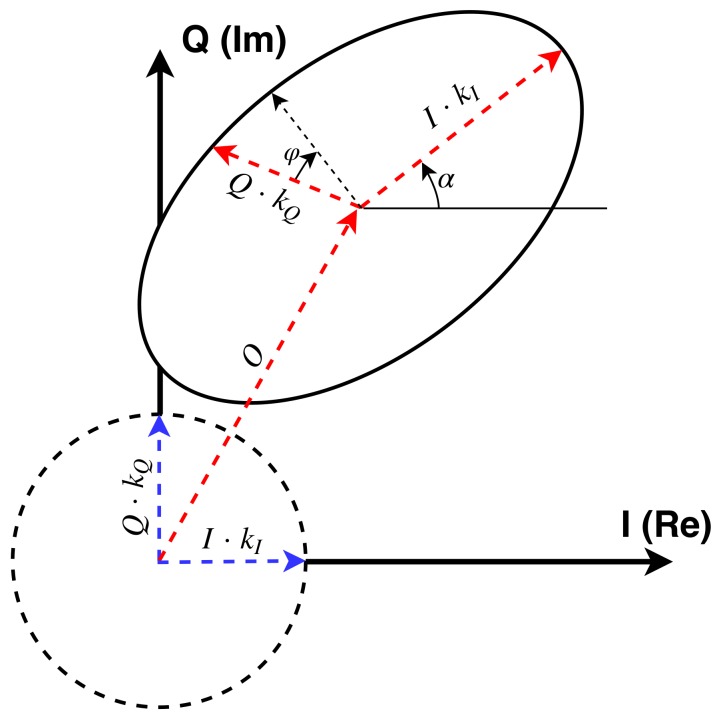
Ideal gain circle (dashed line), and modeled gain ellipsoid (solid line) in the IQ gain plane.

The achieved gain can be represented in the I-Q plane (Figure 8). Ideally, if the values {I,Q}={cos(α),sin(α)} are swept, the achieved gain will be represented as a constant radius circle, centered at the origin, starting at {I,Q}={1,0} over the I axis, and $k_{I}$ and $k_{Q}$ will be orthogonal (blue vectors). Without calibrating, the circle will not be centered (due to the gain offset *O*), the circle will become an ellipsoid (due to the gain unbalance *L*), the gain vectors will not be orthogonal (due to the phase unbalance φ, see red vectors), and will be rotated around the plane origin (due to the phase α). Besides, if we plot the gain circles of all 19 vector modulators of a same beam, they will have different radii (due to different gains *g* in each RF chain). This effects will induce an error in the gain and phase set to any signal.

### 2.3. Beam-Steering Control

The pins of the vector modulators that control the gain of the *I* and *Q* channels require a differential voltage, which is generated using 16-bits differential Digital-to-Analog Converters (DAC). These DACs are controlled using the Inter-Integrated Circuit (I2C) serial protocol and I2C multiplexers, which are controlled by the Linux embedded main controller.

**Figure 9:**
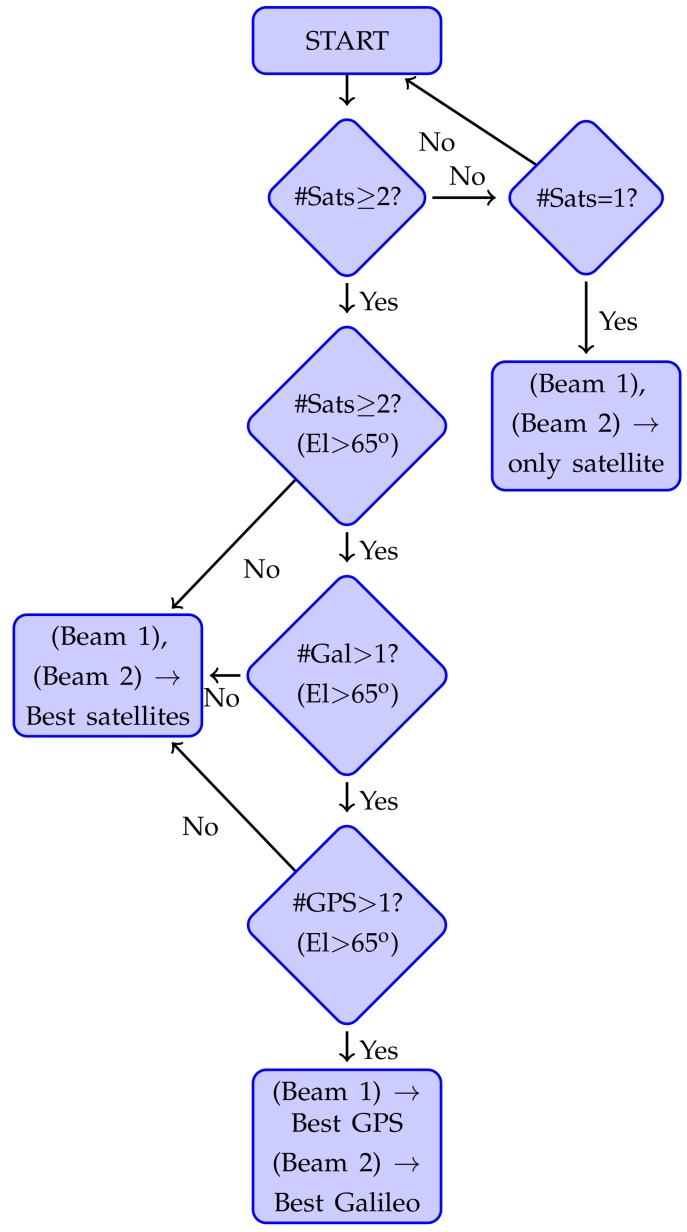
Developed selection algorithm to choose the best satellites available [13].

The required *I* and *Q* gains are computed in near real time with an embedded system [11]. The system first retrieves the available satellites using the Trimble BD982 GNSS Receiver [12] and selects the two optimum available in each frequency band. This GNSS receiver is capable of tracking GPS, GLObalnaya NAvigatsionnaya Sputnikovaya Sistema (GLONASS), Galileo, and Satellite-Based Augmentation Systems (SBAS) in L1/E1, L2, and L5/E5 frequency bands. The satellites are chosen at each frequency band separately following the algorithm described in Figure 9. The system only takes into account satellites whose phase has been locked by the GNSS receiver. In this way, the GNSS receiver provides accurate Doppler frequency, that makes easier the post-processing. It prioritizes to pick one Galileo and one GPS satellites with the highest elevation angle if both elevation angles are over 65${}^{\circ }$. If these conditions are not fulfilled, the system picks the two satellites with the highest elevation angles. The validation of the algorithm was conducted in the laboratory rooftop and the results are shown in Section 4.2.

In order to find where each array has to point, the system first estimates the position of the satellites in ECEF coordinates using the satellite ephemeris [6,14]. Then, the Earth Centered Earth Fixed coordinates (ECEF) of the satellites are transformed to the North-East-Down (NED) local coordinate system, where the satellites are located in the sky with an azimuth and an elevation angle. For the down-looking array, the location on ground to point is determined using a flat Earth model. Once the position of the satellites is known in a local reference plane, the vectors that point to them are rotated using Direction Cosine Matrices (DCM) taking into account both the orientation of the plane and the orientation of the arrays, so the vectors pointing to the satellites are now referenced in a local vehicle-carried coordinate system. If there are enough GNSS satellites in view, the orientation is estimated by the GNSS receiver, which has two antenna input connectors that can be used to determine two Euler angles, and an inclinometer [15], which gives the third rotation angle. If not, a Commercial Off-The-Shelf (COTS) Inertial Measurement Unit [16] is used. Then, the system computes the required *I* and *Q* gains for each vector modulator taking into account the position of the satellites, the position and the orientation of the platform, and the calibration parameters. The system also logs the platform position and attitude, the estimated position error, which satellites are pointed, their approximated Doppler frequency and the pointing angle of each array in the local reference system to later compensate the antenna radiation pattern. The ideal phase of each element is computed as

(5) $$\begin{array}{c} \beta_{m,n}\left(\theta ,\varphi \right)=-d\frac{2\pi }{\lambda }\left(\left(m+n\cdot cos(\gamma )\right)\cdot sin\left(\theta \right)\cdot cos\left(\varphi \right)+n\cdot sin\left(\gamma \right)\cdot sin\left(\theta \right)\cdot sin\left(\varphi \right)\right), \end{array}$$

where *d* is the distance between antennas, λ is the wavelength at the particular frequency band, *m* and *n* are the coordinates of each antenna in the hexagonal grid defined in Figure 5, $\gamma =60^{\circ }$ is the angle between the $\vec{m}$ and $\vec{n}$ vectors in Figure 5, φ is the azimuthal pointing angle defined anti-clockwise from vector $\vec{m}$ to $\vec{n}$, and θ is the off-boresight pointing angle defined from vector $\vec{z}$ to the antenna array ground plane.

Then, if a gain *G* and phase β must be set to a particular branch, first the non-corrected $I_{I}$ and $Q_{I}$ are found taking into account the gain *g* and phase α of that particular RF branch:

(6) $$\begin{array}{c} I_{I}=\frac{|\frac{\sqrt{G}}{g}|}{\sqrt{1+tan^{2}\left(\beta -\alpha \right)}}, \end{array}$$

(7) $$\begin{array}{c} Q_{I}=I_{I}\cdot tan\left(\beta -\alpha \right). \end{array}$$

Then, the *L* and φ parameters are compensated, and the corrected $I_{C}$ and $Q_{C}$ are obtained:

(8) $$\begin{array}{c} Q_{C}=\frac{Q_{I}}{L\cdot \cos\varphi }, \end{array}$$

(9) $$\begin{array}{c} I_{C}=I_{I}-L\cdot \sin\varphi \cdot Q_{C}. \end{array}$$

The calibration parameters *g*, α, *L*, and φ are obtained following the procedure described in Section 3.1. If any offset in the *I* or *Q* components $O_{I}$ and $O_{Q}$ has to be compensated, it has to be removed from $I_{C}$ and $Q_{C}$. These two corrected gains are then written to the DACs using I2C protocol.

### 2.4. Data Sampling and Processing

For the data sampling, 4 dual-input Software Defined Radios (SDR) model Ettus Research Universal Software Radio Peripheral (USRP) X310 are used [17]. Each one samples synchronously a pair of beams. They are synchronized between them using a 1-PPS signal and a 10 MHz reference signal, both generated with an Octoclock [18] which uses for that purpose a GPS-Disciplined Oscillator (GPS-DO). The signals to be sampled are down-converted and sampled at 32.736 Msps and 1 bit for both the *I* and *Q* components of the signal, and then stored in a computer for post-processing.

### 2.5. Calibration System

**Figure 10:**
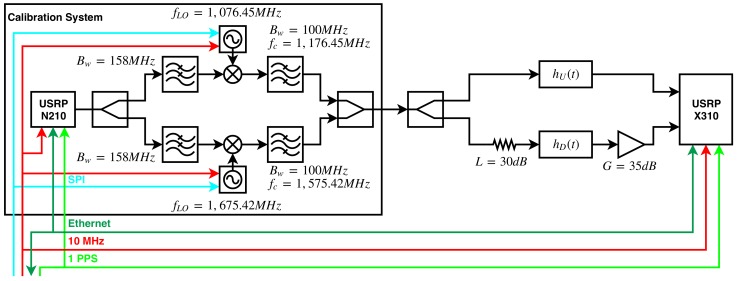
Block diagram of the calibration system. $f_{LO}$ is the frequency of the local oscillator (LO), $B_{w}$ is the −3 dB bandwidth, $f_{c}$ is the −3 dB cut-off frequency, *L* stands for attenuation, and *G* stands for gain.

**Figure 11:**
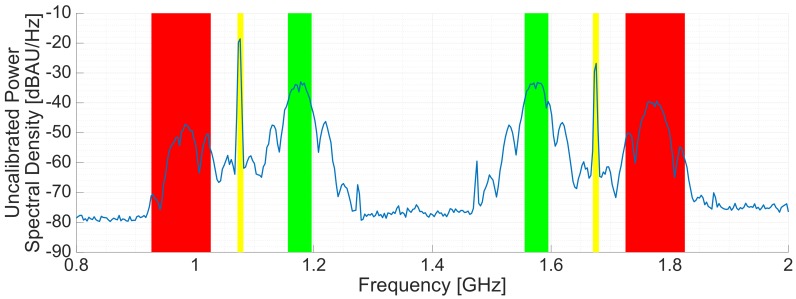
Measured spectra of the transmitted calibration signal at L1/E1 frequency band (**right**), and at L5/E5a frequency band (**left**). In green, the region of the spectrum that will be used for calibration. The aliases of the up-conversion are highlighted in red. The local oscillator leakages are highlighted in yellow.

The instrument calibration is conducted in two steps, the first one in an anechoic chamber, and the second one with a calibration system embedded in the instrument. This calibration system uses a periodic signal made of a concatenated sequence of 10 L5 PRN codes (10 × 10230 chips). The codes are generated in another SDR (URSP N210) at a 10 MHz chiprate (20 MHz bandwidth), with a total sequence length of $T_{c}=$ 10 ms (the last 2300 chips are removed), and a repetition period of $T_{c}$. The signal is generated at Intermediate Frequency IF = 100 MHz and then up-converted at $f_{L1}$ = 1.57542 GHz and at $f_{L5}$ = 1.17645 GHz (Figure 10). The transmitting SDR, the receiving SDR and the local oscillators (LO) used for the up-conversion have all a common 10 MHz reference. The transmitting and receiving devices are also synchronized using a 1 PPS signal. The calibration signal is injected in the up-looking array $h_{U}\left(t\right)$ and in the down-looking array $h_{D}\left(t\right)$. Since the down-looking chain has 35 dB extra gain and the calibration signal is over the noise (Figure 11), a 30 dB attenuator is placed before the down-looking array calibration port to avoid the saturation of the data sampling system during the calibration.

Figure 11 shows the spectra of the calibration signal around the L1/E1 frequency band (left) and the L5/E5a frequency band (right). The aliases of the up-conversion (in red), and the LO leakage (yellow) can be clearly seen in both figures. The region of the spectra that will be used to calibrate the instrument is highlighted in green. The PRN codes were generated at the highest IF possible in order to distance as much as possible the up-conversion aliases and the LO leakage respect to the useful frequency band.

Section 3 digs into the two-step calibration procedure.

### 2.6. Instrument Integration

**Figure 12:**
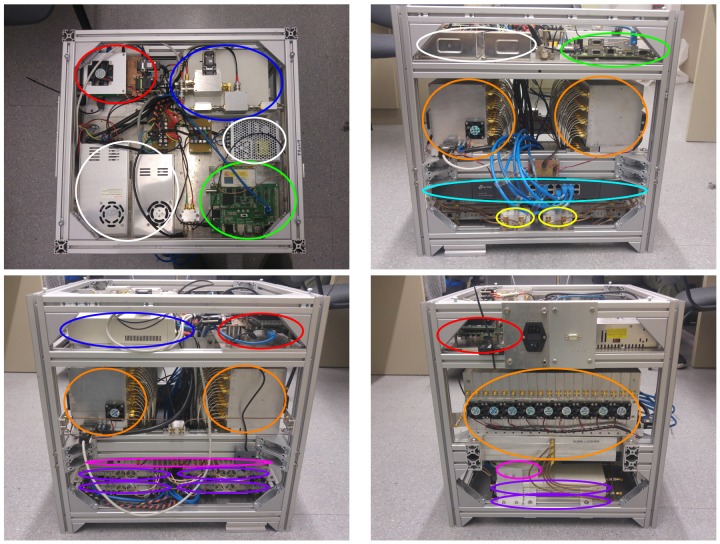
MIR rack. The top floor of the rack has the embedded system (red), the power supplies (white), the calibration system (blue), and the GNSS receiver (green). The middle floor has the beamformers (orange). The lower floor has the Octoclock (magenta), the SDR (purple), the gigabit ethernet swithc (cyan), and the extra amplifiers for the down-looking RF chain (yellow).

All blocks except the arrays, the inclinometer, the GNSS antennas and the IMU are mounted inside a custom-made rack with three different floors (Figure 12) that measures 570 × 500 × 470 mm and weights 70 kg approximately. The top level has the GNSS receiver, the calibration system, all power supplies, and the Linux embedded system. The beamformers are attached in the middle level. There, the 19 cables coming from the arrays are connected to the beamformers. Additionally, there is an extra connector for the cable that connects the calibration system and that array (Figure 12, bottom right). From each beamformer, the 4 output cables go to the lower floor. The cables from the up-looking beamformer are connected directly to the SDRs. The cables of the down-looking beamformers are first connected to RF amplfiers. The bottom level has attached the gigabit ethernet switch, the Octoclock, the extra RF amplifiers for the down-looking RF chain, and the SDR. The inclinometer is attached to one of the sides of the rack, while the inertial measurement unit and the GNSS antennas are attached to the up-looking array.

## 3. Instrument Calibration

The calibration procedure is divided in two steps, a pre-calibration in an anechoic chamber, and the in-flight calibration.

### 3.1. Pre-Calibration

In the pre-calibration, the arrays and the beamformers are characterized. They do not need to be calibrated every time the instrument is turned on, but they should be calibrated from time to time. The pre-calibration has four steps. First, the array and the beamformers are characterized in the anechoic chamber using an externally transmitted signal that is received by the antennas. Then, they are characterized again injecting a signal through the calibration port. Next, from these two measurements, the difference between both paths is estimated, which will be called the “compensation parameters”. Last, once the instrument has been finished and all the final hardware is mounted, it is calibrated again through the calibration port using a vector network analyzer and the “compensation parameters”.

#### 3.1.1. Step 1: Calibration through the Antenna

**Figure 13:**
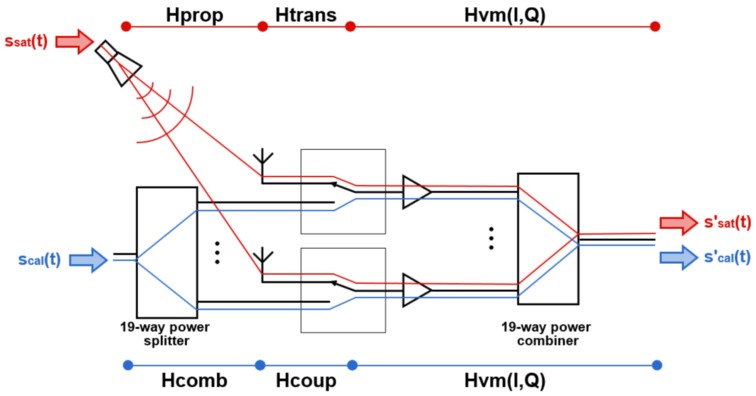
Received and calibration signal’s paths to the receiver in one of the bands and beams.

The received signals from the satellites follow the red path in Figure 13. The signal $s_{sat}$ is propagated from the satellite (or the transmitting antenna inside the anechoic chamber) to the phase center of the antenna ($H_{prop_{i}}=g_{prop_{i}}\cdot e^{-j\alpha_{prop_{i}}}$), passes through the calibration switch ($H_{trans_{i}}=g_{trans_{i}}\cdot e^{-j\alpha_{trans_{i}}}$) and through the vector modulator $H_{VM_{i}}\left(I,Q\right)=I\cdot k_{I_{i}}+Q\cdot k_{Q_{i}}+O_{i}$. The two last contributions $H_{trans_{i}}$ and $H_{VM_{i}}$ are the ones that must be calibrated. To conduct these measurements, an external antenna is required. In the MIR case, it was mounted in an anechoic chamber. The transmitting antenna was a reference antenna connected to the transmitting port of a vector network analyzer, whereas the receiving port was connected to the output of the beamformer.

The outcoming signal $s_{sat}^{\prime }\left(t\right)$ is

(10) $$\begin{array}{cc} s_{sat}^{\prime }\left(t,I_{1},Q_{1},\ldots ,I_{19},Q_{19}\right) & =\sum_{i=1}^{19}s_{sat}\left(t\right)\cdot H_{prop_{i}}\cdot H_{trans_{i}}\cdot H_{VM_{i}}\left(I_{i},Q_{i}\right)\\ & =\sum_{i=1}^{19}s_{sat}\left(t\right)\cdot H_{prop_{i}}\cdot H_{trans_{i}}\cdot \left(I_{i}\cdot k_{I_{i}}+Q_{i}\cdot k_{Q_{i}}+O_{i}\right)=s_{sat}\left(t\right)\cdot H_{n}, \end{array}$$

where $H_{n}$ is the transfer function measured by the VNA. In case one single vector modulator *i* has to be characterized, the rest of gains can be set to zero

(11) $$\begin{array}{cc} s_{sat_{i}}^{\prime }\left(t,0,0,\ldots ,I_{i},Q_{i},\ldots ,0,0\right) & =s_{sat}\left(t\right)\cdot H_{prop_{i}}\cdot H_{trans_{i}}\cdot \left(I_{i}\cdot k_{I_{i}}+Q_{i}\cdot k_{Q_{i}}\right)+s_{sat}\left(t\right)\cdot \sum_{i=1}^{19}H_{prop_{i}}\cdot H_{trans_{i}}\cdot O_{i}\\ & =s_{sat}\left(t\right)\cdot H_{prop_{i}}\cdot H_{trans_{i}}\cdot \left(I_{i}\cdot k_{I_{i}}+Q_{i}\cdot k_{Q_{i}}\right)+s_{sat}\left(t\right)\cdot O_{T}. \end{array}$$

As it can be seen, the offset contribution is always present and constant. In order to estimate the calibration parameters *g*, α, *L*, and φ, it is required to estimate the vectors $k_{I}$, $k_{Q}$, and *O* in Equation (4). For each vector modulator *i*, several transfer functions $H_{n}$ are measured setting *N* different pairs of $\left\{I_{n},Q_{n}\right\}$ values. The following linear system is obtained:

(12) $$\begin{array}{c} A\cdot \vec{x}=\left(\begin{array}{ccc} I_{1} & Q_{1} & 1\\ \ldots & \ldots & \ldots \\ I_{n} & Q_{n} & 1\\ \ldots & \ldots & \ldots \\ I_{N} & Q_{N} & 1 \end{array}\right)\cdot \left(\begin{array}{c} H_{prop_{i}}\cdot H_{trans_{i}}\cdot k_{I_{i}}\\ H_{prop_{i}}\cdot H_{trans_{i}}\cdot k_{Q_{i}}\\ O_{T} \end{array}\right)=\left(\begin{array}{ccc} I_{1} & Q_{1} & 1\\ \ldots & \ldots & \ldots \\ I_{n} & Q_{n} & 1\\ \ldots & \ldots & \ldots \\ I_{N} & Q_{N} & 1 \end{array}\right)\cdot \left(\begin{array}{c} k_{I_{i}}^{\prime }\\ k_{Q_{i}}^{\prime }\\ O_{T} \end{array}\right)=\left(\begin{array}{c} H_{1}\\ \ldots \\ H_{n}\\ \ldots \\ H_{N} \end{array}\right)=\vec{H}. \end{array}$$

If more than three independent measurements are used, the system can be solved using the linear least squared method

(13) $$\begin{array}{c} \hat{x}={\left(A^{T}\cdot A\right)}^{-1}\cdot A^{T}\cdot \vec{H}. \end{array}$$

This linear approximation requires a set of $\left\{I_{n},Q_{n}\right\}$ values that maintain the RF devices in the linear region. The MIR instrument uses

(14) $$\begin{array}{c} \left\{I_{n},Q_{n}\right\}=\left\{\cos\frac{n}{N}\cdot 360^{\circ },\sin\frac{n}{N}\cdot 360^{\circ }\right\},\;\forall n\in N:\left\{1\ldots N\right\}\\ \left\{I_{N+1},Q_{N+1}\right\}=\left\{0,0\right\}, \end{array}$$

with N=8. Once the system is solved, the $H_{prop_{i}}$ contribution can be removed

(15) $$\begin{array}{cc} H_{prop_{i}} & =\frac{1}{|\vec{r_{i}}|}\cdot e^{-j\cdot \frac{2\pi }{\lambda }\cdot \left|\vec{r_{i}}\right|}, \end{array}$$

where $\vec{r_{i}}$ is the vector between the phase centers of the transmitting and the *i* receiving antenna, and λ is the wavelength.

Then, the calibration parameters (*g* and α), and the unbalance parameters of the vector modulator (*L* and φ) of each RF chain are obtained from $\vec{I_{i}^{\prime }}$ and $\vec{Q_{i}^{\prime }}$ as:

(16) $$\begin{array}{cc} g_{i} & =|k_{{}_{i}^{\prime }}^{\prime }|, \end{array}$$

(17) $$\begin{array}{cc} \alpha_{i} & =-∡\{k_{I_{i}}^{\prime }\}, \end{array}$$

(18) $$\begin{array}{cc} L_{i} & =\frac{|k_{Q_{i}}^{\prime }|}{|k_{I_{i}}^{\prime }|}, \end{array}$$

(19) $$\begin{array}{cc} \varphi_{i} & =∡\left\{k_{I_{i}}^{\prime }\right\}-∡\left\{k_{Q_{i}}^{\prime }\right\}+\frac{\pi }{2}. \end{array}$$

Equation (11) shows that it is not possible to separate the contributions of the different offsets, but the aggregated offset can be compensated in a single Vector Modulator chain *k*. To do so, the offsets $O_{T_{I}}$ and $O_{T_{Q}}$ must be subtracted from the $I_{C}$ and $Q_{C}$ in Equation (9) of the chosen element:

(20) $$\begin{array}{c} O_{T_{I}}\cdot k_{I_{k}}+O_{T_{Q}}\cdot k_{Q_{k}}=\sum_{i=1}^{19}H_{prop_{i}}\cdot H_{trans_{i}}\cdot O_{i}=O_{T}\\ O_{T_{I}}=k_{I_{k}}\cdot \frac{O_{T}}{|O_{T}|},\;O_{T_{Q}}=k_{Q_{k}}\cdot \frac{O_{T}-O_{T_{I}}\cdot k_{I_{k}}}{|O_{T}-O_{T_{I}}\cdot k_{I_{k}}|} \end{array}$$

#### 3.1.2. Step 2: Calibration through the Calibration Port

Since it is unpractical to periodically calibrate the instrument with an external antenna, even more if the instrument is mounted in an airplane, the instrument has a calibration port in each array (blue path in Figure 13). There, the calibration signal $s_{cal}\left(t\right)$ is split and distributed to the 19 RF Front-ends ($H_{comb_{i}}=g_{comb_{i}}\cdot e^{-j\alpha_{comb_{i}}}$). The calibration signal is coupled to the RF main line due to the switch finite isolation ($H_{coup_{i}}=g_{coup_{i}}\cdot e^{-j\alpha_{coup_{i}}}$), and then the signal follows the same route than the signal coming from the antennas through the rest of the RF chain ($H_{VM_{i}}$).

To conduct these measurements, the transmitting port of the anechoic chamber’s VNA was connected to the calibration port, whereas the receiving port was connected to the output of the beamformer. In the MIR case it was done just after the first calibration step, connecting to the calibration port the cable that fed the transmitting antenna.

The outgoing signal $s_{cal}^{\prime }\left(t\right)$ is in this case

(21) $$\begin{array}{cc} s_{cal}^{\prime }\left(t,I_{1},Q_{1},\ldots ,I_{19},Q_{19}\right) & =\sum_{i=1}^{19}s_{cal}\left(t\right)\cdot H_{comb_{i}}\cdot H_{coup_{i}}\cdot H_{VM_{i}}\left(I_{i},Q_{i}\right)\\ & =\sum_{i=1}^{19}s_{cal}\left(t\right)\cdot H_{comb_{i}}\cdot H_{coup_{i}}\cdot \left(I_{i}\cdot k_{I_{i}}+Q_{i}\cdot k_{Q_{i}}+O_{i}\right)=s_{cal}\left(t\right)\cdot H_{n}. \end{array}$$

Again, the system of Equation (12) has to be solved, but in this case the vector $\vec{x}$ is

(22) $$\begin{array}{c} \vec{x}=\left(\begin{array}{c} H_{comb_{i}}\cdot H_{coup_{i}}\cdot k_{I_{i}}\\ H_{comb_{i}}\cdot H_{coup_{i}}\cdot k_{Q_{i}}\\ O_{T} \end{array}\right)=\left(\begin{array}{c} k_{I_{i}}^{\prime \prime }\\ k_{Q_{i}}^{\prime \prime }\\ O_{T} \end{array}\right). \end{array}$$

The calibration parameters in Equations (16)–(19) are then computed with $k_{I_{i}}^{\prime \prime }$ and $k_{Q_{i}}^{\prime \prime }$.

#### 3.1.3. Step 3: Obtaining the Compensation Parameters

As aforementioned, the calibration through the calibration port is more practical than the calibration with an external reference antenna, but the calibration signal does not follow the same path that the signals that come from the satellites. It is proposed to measure the differences in the paths, so this path difference can be compensated in the calibration parameters obtained through the calibration port. From Figure 13, they can be defined as

(23) $$\begin{array}{c} H_{comp_{i}}=\frac{H_{sat_{i}}\left(I_{i},Q_{i}\right)}{H_{cal_{i}}\left(I_{i},Q_{i}\right)\cdot H_{prop_{i}}}=\frac{H_{trans_{i}}}{H_{comb_{i}}\cdot H_{coup_{i}}}. \end{array}$$

Considering that they depend on a particular pair of $\left\{I_{i},Q_{i}\right\}$ values, it is instead proposed to compute them as

(24) $$\begin{array}{c} H_{comp_{i}}=\frac{k_{I_{i}}^{\prime }}{H_{prop_{i}}\cdot k_{I_{i}}^{\prime \prime }}=\frac{\left(H_{prop_{i}}\cdot H_{trans_{i}}\cdot k_{I_{i}}\right)}{H_{prop_{i}}\cdot \left(H_{comb_{i}}\cdot H_{coup_{i}}\cdot k_{I_{i}}\right)}=\frac{H_{trans_{i}}}{H_{comb_{i}}\cdot H_{coup_{i}}}. \end{array}$$

Note that the compensation parameters do not depend on the vector modulators, and therefore they will not change between beams of the same band.

#### 3.1.4. Step 4: Re-Calibrating the Instrument

If any hardware after the calibration switch has changed or the instrument needs to be calibrated again, this can be done through the calibration port and then applying the “compensation parameters”. To do so, the instrument has to be first calibrated following the step 2 in Section 3.1.2 to obtain a new pair of $\left\{{\hat{k^{\prime \prime }}}_{i},{\hat{k^{\prime \prime }}}_{i}\right\}$, and then they are multiplied by the compensation parameter $H_{comp_{i}}$

(25) $$\begin{array}{cc} {\hat{k_{I}}}_{i}^{\prime \prime }\cdot H_{comp_{i}} & ={\hat{k_{I}^{\prime }}}_{i} \end{array}$$

(26) $$\begin{array}{cc} {\hat{k_{Q}}}_{i}^{\prime \prime }\cdot H_{comp_{i}} & ={\hat{k_{Q}^{\prime }}}_{i}\;\;, \end{array}$$

where ${\hat{k_{I}}}_{i}^{\prime }$ and ${\hat{k_{Q}}}_{i}^{\prime }$ must be used to compute the calibration parameters as explained in Equations (16)–(19). After that, the offsets in Equation (20) should be recalculated.

### 3.2. In-Flight Calibration

In the in-flight calibration it is estimated the random phase of the local oscillators for later compensation. Besides, during the pre-calibration, the gain of the elements was calibrated with respect to the elements of the same array, so it is required to perform a calibration between both arrays. To do so, a calibration signal is injected as explained in Section 3.2, and it is corrected in post-processing. In the MIR case, the power splitter and the cables that connect the calibration system to the calibration port were not pre-calibrated, so it they were characterized for later compensation.

In this step only one measurement per USRP is required to characterize the phase and gain between the up and down-looking channels. If a known gain *G* and phase β are set to one element in the up and down-looking arrays for the same beam (that is, the same USRP), the phase and gain differences between the sampled signals is the phase and gain differences between channels. To estimate these differences a calibration signal is injected in the arrays and sampled with the USRPs for later processing and compensation.

The up-converted signal is injected in each RF Front-end. All vector modulators except the one corresponding to the central element of the array are disabled. The outcoming is sampled with the receiving SDRs. As for the normal operation mode, the calibration signals are sampled by beam pairs. In the calibration mode, however, the receiving USRP samples the signals at 10 Msps with 16 bits for the *I* and *Q* components. The sampled signal y(t) is

(27) $$\begin{array}{c} y\left(t\right)=\left\{\left[\left(x_{T}\left(t\right)\cdot e^{-j\left(2\pi f_{TX}t+\phi_{TX}\right)}\right)*\left(h_{TX}\left(t\right)\cdot e^{-j2\pi f_{c}t}\right)\right]*\left(h\left(t\right)\cdot e^{-j2\pi f_{c}t}\right)+n\left(t\right)\cdot e^{-j2\pi f_{c}t}\right\}\cdot \\ e^{j\left(2\pi f_{RX}t+\phi_{RX}\right)}, \end{array}$$

where $x_{T}\left(t\right)=\sum_{n}x\left(t-n\cdot T_{code}\right)$ is the $T_{code}$ periodic base-band transmitted calibration signal, $f_{TX}$ is the up-conversion frequency, $\phi_{TX}$ is the random phase of the transmitting local oscillator, $h_{TX}$ is the equivalent base-band impulse response of the transmitting USRP, * is the convolution operator [19], *h* is the combined equivalent base-band impulse response of the Device Under Test (DUT), the splitting network after the calibration system, and the receiving USRP, $f_{c}$ is the band central frequency, n(t) is the base-band additive white Gaussian noise present in the channel, $f_{RX}$ is the down-conversion frequency, and $\phi_{RX}$ is the random phase of the receiving local oscillator. If the transfer functions of $h_{TX}\left(t\right)$ and h(t) are flat enough to state that $H\left(f_{c}\right)\approx H\left(f_{TX}\right)$, $f_{c}=f_{TX}$ can be approximated to simplify Equation (27)

(28) $$\begin{array}{cc} y(t) & =\left\{\left[x_{T}\left(t\right)*h_{TX}\left(t\right)*h\left(t\right)+n\left(t\right)\right]\cdot e^{-j\left(2\pi f_{TX}t+\phi_{TX}\right)}\right\}\cdot e^{j\left(2\pi f_{RX}t+\phi_{RX}\right)}\\ & =\left[x_{T}\left(t\right)*h_{TX}\left(t\right)*h\left(t\right)+n\left(t\right)\right]\cdot e^{-j\left(2\pi \Delta ft+\Delta \phi \right)}=\left[x_{T}\left(t\right)*h^{\prime }\left(t\right)\right]\cdot e^{-j\left(2\pi \Delta ft+\Delta \phi \right)}+n^{\prime }\left(t\right), \end{array}$$

where $\Delta f=f_{RX}-f_{TX}$ and $\Delta \phi =\phi_{RX}-\phi_{TX}$ are the differences between the frequencies and the random phases of the local oscillators in the up and down conversion respectively, and $n^{\prime }\left(t\right)=n\left(t\right)\cdot e^{-j\left(2\pi \Delta ft+\Delta \phi \right)}$, and $h^{\prime }\left(t\right)=h_{TX}\left(t\right)*h\left(t\right)$ have been abbreviated for simplicity.

First, the residual frequency difference between oscillators has to be compensated, otherwise the phase will not remain constant over time. To do so, y(t) is circularly cross-correlated with a $T_{code}$ length clean replica of the calibration signal x(t). Then, the phase in the cross-correlation peak is used to estimate the frequency error between oscillators, as it is proven in Equation (A1) from Appendix A

(29) $$\begin{array}{c} R_{yx}\left(t,\tau ,\Delta f\right)=\left(\left(h^{\prime }\left(t\right)\cdot e^{-j2\pi \Delta ft}\right)\overset{t}{*}\left(\sum Y\left(t-nT_{code},\Delta f\right)\cdot e^{j2\pi \Delta fnT_{c}}\right)\right)\left(\tau ,\Delta f\right)\cdot e^{-j\Delta \phi }+R_{n^{\prime }x}\left(t,\tau \right), \end{array}$$

where $T_{code}$ is the time length of the code, $T_{c}$ is the chip duration, $\overset{t}{*}$ is the convolution in the time domain, and Y(τ,Δf) is the Woodward Ambiguity Function (WAF) which for Binary Phase Shifting Keying (BPSK) codes can be approximated as [20]

(30) $$\begin{array}{c} Y\left(\tau ,f\right)=▵\left(\frac{\tau }{T_{c}}\right)\cdot sinc\left(f\cdot T_{coh}\right), \end{array}$$

where $T_{coh}$ is the coherent integration time, and

(31) $$\begin{array}{c} ▵\left(\frac{\tau }{T_{c}}\right)=\left\{\begin{array}{cc} 1-\frac{\left|\tau \right|}{T_{c}}, & \;|\tau |<T_{c},\\ 0, & \;\mathrm{elsewhere}. \end{array}\right. \end{array}$$

If a simplified model of the transmitting impulse response $h_{TX}\left(t\right)=A_{TX}\cdot \delta \left(t-\tau_{TX}\right)\cdot e^{j\phi_{TX}}$ is assumed, so that the channel behaves as a simplified model $h\left(t\right)=A_{h}\cdot \delta \left(t-\tau_{h}\right)\cdot e^{j\phi_{h}}$, so $h^{\prime }\left(t\right)=A_{TX}\cdot A_{h}\cdot \delta \left(t-\tau_{TX}-\tau_{h}\right)\cdot e^{j(\phi_{TX}+\phi_{h})}=A_{c}\cdot \delta \left(t-\tau_{c}\right)\cdot e^{j\phi_{c}}$, according to Equation (A5) from Appendix A, Equation (29) becomes

(32) $$\begin{array}{cc} R_{yx}\left(t,\tau ,\Delta f\right) & =A_{c}\cdot \left(\sum ▵\left(\frac{\tau -\tau_{c}-nT_{code}}{T_{c}}\right)\cdot sinc\left(\Delta f\cdot T_{c}\right)\cdot e^{j2\pi \Delta fnT_{c}}\right)\cdot e^{-j(2\pi \Delta f\tau_{c}+\Phi_{c}+\Delta \phi )}+R_{n^{\prime }x}\left(t,\tau \right). \end{array}$$

The correlation peaks are located at $\tau =\tau_{c}+nT_{code}$, so that $\tau_{c}$ can be found by correlating the first $T_{c}$ of the sampled data. Then, the peak value is

(33) $$\begin{array}{cc} R_{yx}\left(t,\tau =\tau_{c}+nT_{code},\Delta f\right) & =A_{c}\cdot sinc\left(\Delta f\cdot T_{c}\right)\cdot e^{j2\pi \Delta fnT_{code}}\cdot e^{-j(2\pi \Delta f\tau_{c}+\Phi_{c}+\Delta \phi )}+R_{n^{\prime }x}\left(t,\tau_{c}+nT_{code}\right). \end{array}$$

The phase rotates due to the term $e^{j2\pi \Delta fnT_{c}}$. If the SNR is high enough, the phase can be used to estimate the term Δf, and once the frequency error can be compensated in a similar way [21]. When the sampled signal is correlated in intervals of $T_{coh}$ with the clean replica of the code, the spectrum of each interval is

(34) $$\begin{array}{cc} S_{yx}\left(f\right) & =\left(X\left(f\right)\cdot H_{TX}\left(f\right)\cdot H\left(f\right)+N\left(f\right)\right)\cdot e^{-j\Delta \phi }\cdot X^{*}\left(f\right)\\ & =X\left(f\right)\cdot X^{*}\left(f\right)\cdot H_{TX}\left(f\right)\cdot H\left(f\right)\cdot e^{-j\Delta \phi }+N\left(f\right)\cdot X^{*}\left(f\right)\cdot e^{-j\Delta \phi }\\ & ={\left|X\left(f\right)\right|}^{2}\cdot H_{TX}\left(f\right)\cdot H\left(f\right)\cdot e^{-j\Delta \phi }+N\left(f\right)\cdot X^{*}\left(f\right)\cdot e^{-j\Delta \phi }\\ & ={\left|X\left(f\right)\right|}^{2}\cdot |H_{TX}\left(f\right)|\cdot e^{-j∡H_{TX}\left(f\right)}\cdot \left|H\left(f\right)\right|\cdot e^{-j∡H(f)}\cdot e^{-j\Delta \phi }+N\left(f\right)\cdot X^{*}\left(f\right)\cdot e^{-j\Delta \phi }. \end{array}$$

In order to retrieve the modulus of the combined transfer functions, the spectra contribution of the PRN codes ${\left|X\left(f\right)\right|}^{2}$ has to be removed. The Fourier transforms of the PRN codes are known and exhibit null values. Dividing $S_{yx}$ by the code spectra ${\left|X\left(f\right)\right|}^{2}$ will therefore result in an inaccurate estimation of the transfer functions H(f) and $H_{TX}\left(f\right)$. It is therefore recommended to estimate $S_{yx}$ and ${\left|X\left(f\right)\right|}^{2}$ using the Barlett’s method [19]. To do so, each $T_{code}$ interval of the *K* intervals of the input signal y(t) is divided into *M* sub-intervals. The optimum number of sub-intervals to reduce the variance of the estimation of all three parameters was found empirically to be M=500 for the MIR instrument. Knowing the delay $\tau_{c}$, these sub-intervals are cross-correlated with the corresponding part of the transmitted code. The *M* sub-intervals are then averaged to get a smoothed estimation of the spectra $|X_{k}{\left(f\right)|}^{2}$, which is used to estimate the transfer functions in Equation (35). Last, the *K* estimated transfer functions are averaged to reduce the impact of the noise W(f).

The estimated transfer function $H_{yx}\left(f\right)$ of the up-looking and down-looking channels becomes:

(35) $$\begin{array}{cc} H_{y_{u}x}\left(f\right) & =|H_{TX}\left(f\right)|\cdot e^{-j∡H_{TX}\left(f\right)}\cdot \left|H_{u}\left(f\right)\right|\cdot e^{-j∡H_{u}\left(f\right)}\cdot e^{-j\Delta \phi_{u}} \end{array}$$

(36) $$\begin{array}{cc} H_{y_{d}x}\left(f\right) & =|H_{TX}\left(f\right)|\cdot e^{-j∡H_{TX}\left(f\right)}\cdot \left|H_{d}\left(f\right)\right|\cdot e^{-j∡H_{d}\left(f\right)}\cdot e^{-j\Delta \phi_{d}}, \end{array}$$

where the subscripts *u* and *d* stand for the up- and down-looking channels, respectively. By dividing both transfer functions we obtain

(37) $$\begin{array}{cc} H_{ud}\left(f\right) & =\frac{|H_{u}\left(f\right)|\cdot e^{-j∡H_{u}\left(f\right)}\cdot e^{-j\Delta \phi_{u}}}{|H_{d}\left(f\right)|\cdot e^{-j∡H_{d}\left(f\right)}\cdot e^{-j\Delta \phi_{d}}}=\frac{|H_{u}\left(f\right)|}{|H_{d}\left(f\right)|}\cdot e^{-j(∡H_{u}\left(f\right)-∡H_{d}\left(f\right))}\cdot e^{-j(\Delta \phi_{u}-\Delta \phi_{d})}, \end{array}$$

which is used for the relative calibration between channels. Note that h(t) also includes the contributions of the power splitting network after the calibration system, and they have to be removed. To do so, these parts are measured using a 4-port vector network analyzer (Rohde-Schwarz ZVB-8).

**Figure 14:**
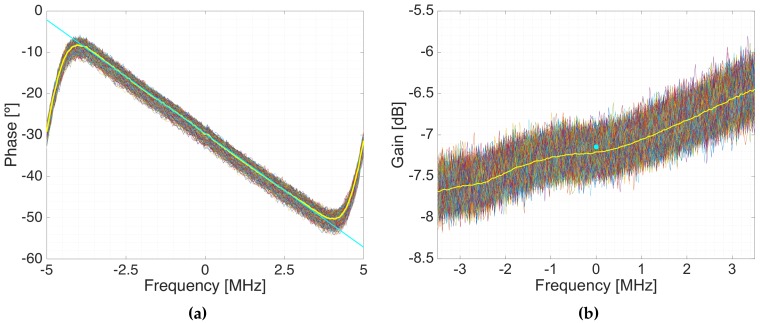
Estimated phase (**a**) and gain (**b**) of the different $T_{code}$ intervals. The time averaged phase response estimation is plot in yellow, and the linear approximation in cyan. A value of M=500 was used. The time geometrically averaged amplitude of the transfer function is plot in yellow, and the geometric average gain is plot as a dot in cyan.

Figure 14 shows the estimated $∡H_{TX}\left(f\right)$ for all $T_{code}$ intervals of one of RF chains using M=500 sub-intervals, and the estimated linearization. As it can be seen, the phase is linear, and the y-intercept points of the different $T_{code}$ intervals are similar, proving that the frequency error between local oscillators is successfully compensated, otherwise the phase (y-intercept point) will be rotating. The estimated phase was −29${}^{\circ }$ with an standard deviation of 0.88${}^{\circ }$. The estimated group delay was 15.26 ns with an standard deviation of 0.11 ns. The estimated gain was −7.14 dB with a geometric standard deviation of 0.1 dB.

**Figure 15:**
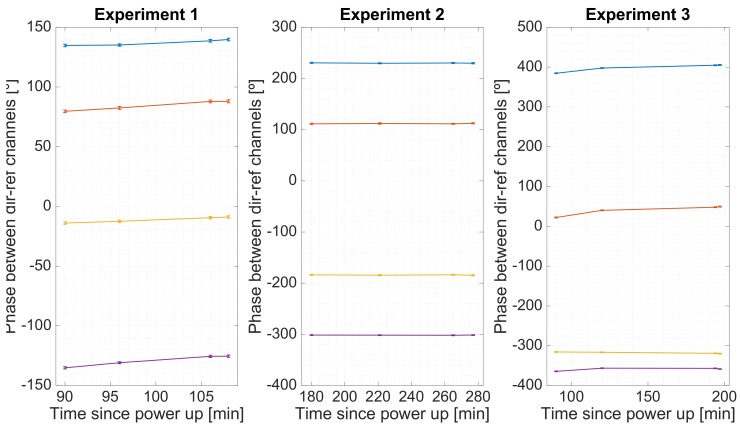
Estimated phase between the direct and reflected RF channels for the four different USRPs during three sets of experiments. The lines show the drift between the estimations, and the markers are error bars.

**Figure 16:**
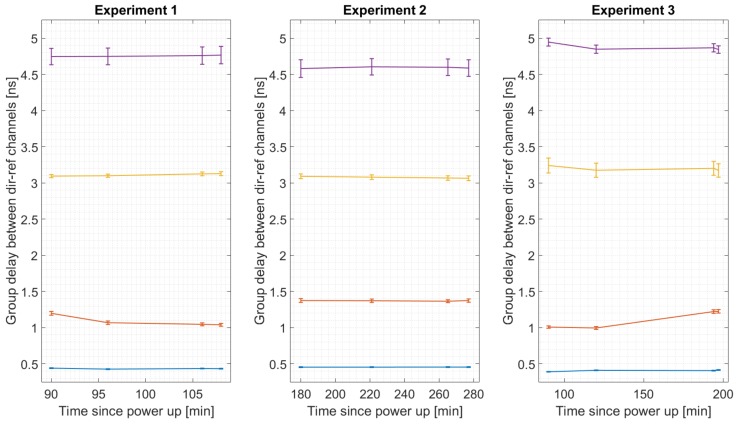
Estimated group delay between the direct and reflected RF channels for the four different USRPs during three sets of experiments. The lines show the drift between the estimations, and the markers are error bars.

**Figure 17:**
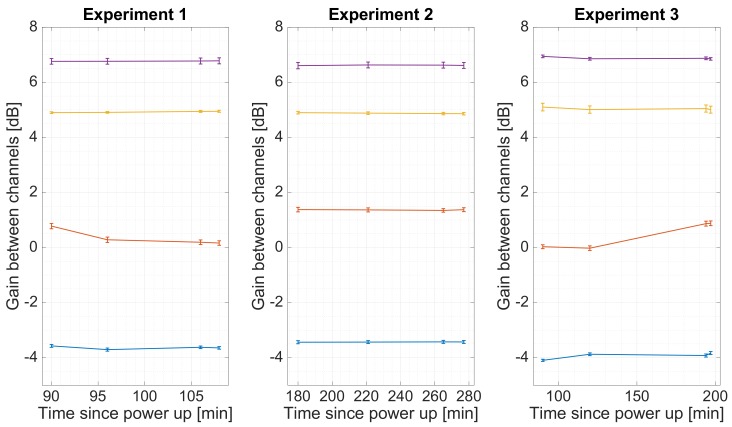
Estimated gain between the direct and reflected RF channels for the four different USRPs during three sets of experiments. The lines show the drift between the estimations, and the markers are error bars. The mean and the standard deviation have been computed as geometric mean and geometric standard deviation.

Three experiments were set to analyze the behavior of the system over time. These 3 experiments were conducted in different winter days in a room without heating, with 4 calibration measurements done in each experiment. Figure 15 shows the estimated phase between the direct and reflected RF chains for the 4 different USRPs (each one represented with a different color). As it can be seen, the phase remains constant, which proves that the frequency error between oscillators has been succesfully corrected. Figure 16 shows the group delay between RF chains, and Figure 17 shows the gain between channels. From these plots can be estimated that more than 150 min were required to heat up the instrument. Note that in a warmer environment the heating up time might be shorter.

## 4. Validation Results

This section describes the main experiments carried out to check that the instrument works properly prior to start any field campaign. First, during the pre-calibration in the anechoic chamber, some radiation patterns were measured, both with the instrument calibrated and uncalibrated, and the beam-steering capabilities were tested. Later on, the instrument was placed in the laboratory rooftop tracking GPS and Galileo satellites to ensure that the instrument is able to receive properly all the desired GNSS signals with proper power levels.

### 4.1. Calibration Impact and Measured Radiation Patterns

**Figure 18:**
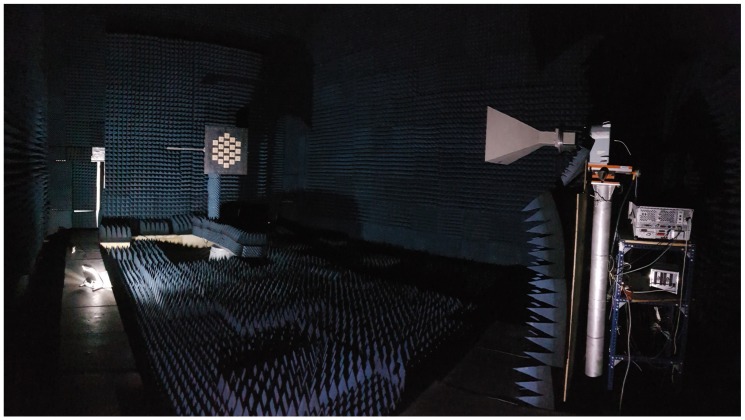
MIR down-looking array being calibrated and measured at the Universitat Politècnica de Catalunya (UPC) anechoic chamber. The 19 elements MIR array is at the back left, while the transmitting reference horn antenna is at the front right. The transmitting antenna has linear polarization and can rotate along the boresight axis.

During the pre-calibration in the anechoic chamber (Figure 18), the beam-steering capabilities of the antenna arrays were also evaluated.

**Figure 19:**
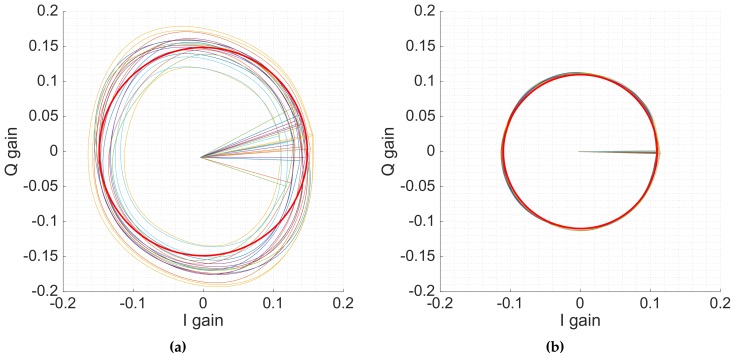
IQ gain circles before (**a**) and after calibration (**b**). The radii lines mark where the circle starts to “rotate”. A red circle with average radius has been added to ease the viewing the effect of the amplitude unbalance.

The beamformers and the arrays were calibrated following the procedure described in Section 3.1. Figure 19 shows the IQ gain circles explained in Section 2.2 of all vector modulators of an L1 beam before (left) and after calibrating (right). A red circle with the average gain has been drawn to ease the viewing of the improvement and the impact of the amplitude unbalance. The radii lines mark the $\alpha_{i}$ parameter, which is where the gain circle starts to “rotate”. Before calibration, when setting a desired angle in the vector modulators, the achieved angle had an Root Mean Square (RMS) error of 13${}^{\circ }$, at L1 and 16${}^{\circ }$ at L5. After calibration, the RMS improves down to 4.9${}^{\circ }$ and 4.2${}^{\circ }$, respectively. Similarly, amplitude errors were corrected from 0.55 dB and 0.11 dB to 0.02 dB and 0.03 dB at L1 and L5 respectively. The approximated impact of these errors in the array directivity are shown in Table 3 [22].

**Figure 20:**
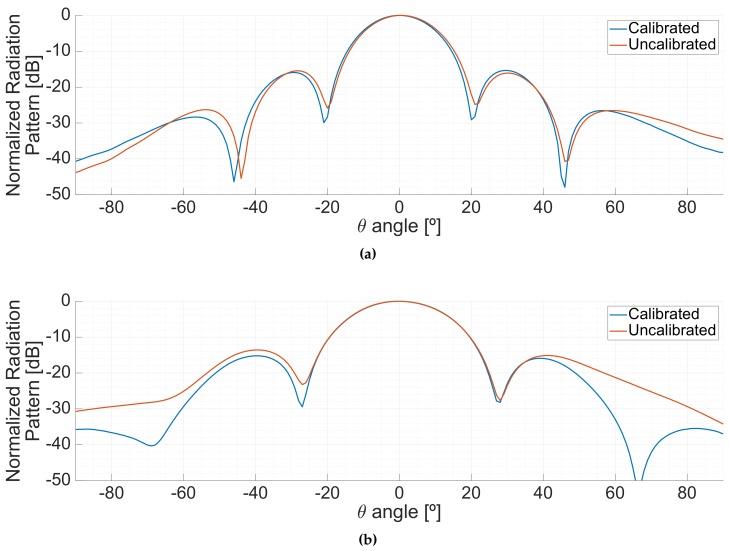
Effect of the calibration in the radiation patterns. Planar cuts in the $\varphi =30^{\circ }$ plane at L1/E1 (**a**) and L5/E5 (**b**).

Figure 20 shows the impact of the calibration in the radiation pattern when the down-looking array (LHCP) was pointing to the boresight of the antenna both at L1/E1 (top) and at L5/E5 (bottom) frequency bands. The calibration improves the nulls of the radiation pattern in both bands. Besides, the uncalibrated beam in L1 is slightly tilted towards the right, which is corrected due to the calibration.

**Figure 21:**
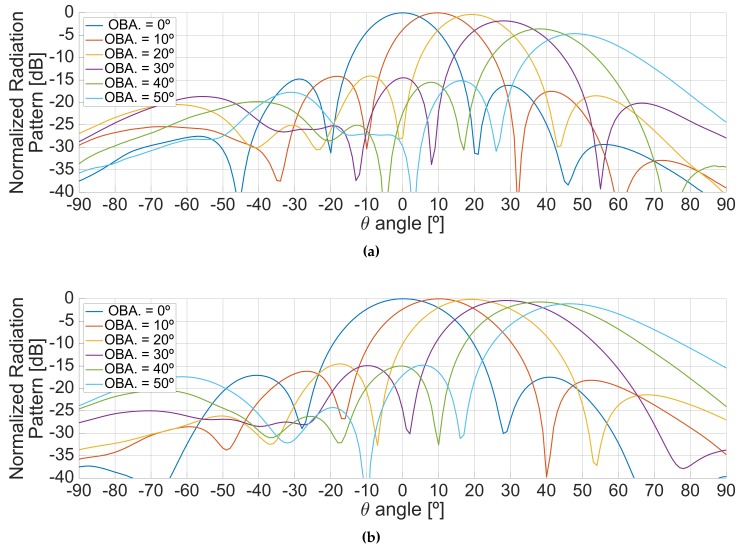
Planar cuts of the radiation pattern along the $\varphi =0^{\circ }$ plane when tilting the beam in the same plane for different off-boresight pointing angles, at the L1/E1 (**a**) and L5/E5 (**b**) frequency bands.

**Figure 22:**
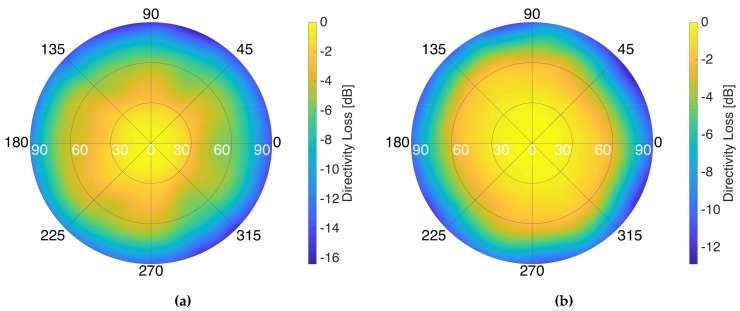
Directivity loss as a function of the pointing direction at L1/E1 (**a**), and at L5/E5 (**b**), for the down-looking array.

After the beamformer calibration, the beam-steering capabilities were tested by tilting the beams from the boresight of the antenna to one of the sides, and some cuts of the antenna radiation pattern were measured. Figure 21 shows the antenna radiation patterns measured in the same plane where the beams were tilted, at L1/E1 (top), and at L5/E5 (bottom) frequency bands. The experiment was carried out for off-boresight angles (OBA) from 0${}^{\circ }$ to 50${}^{\circ }$ in steps of 10${}^{\circ }$. As it can be seen, the maximum decreases as the beam is pointed far from the boresight due to the antenna radiation pattern of the individual elements, and due to the smaller projected area of the array. This loss of directivity was measured as well in order to be compensated while conducting scatterometric measurements and it can be seen in Figure 22. Similarly, the phase dependence to the direction of arrival for altimetric measurements was also measured.

**Figure 23:**
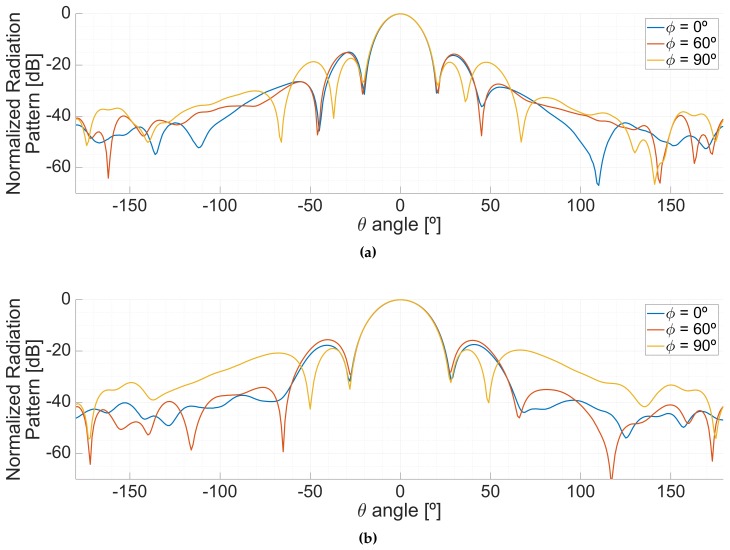
Azimuthal cuts of the full radiation pattern pointing to the boresight of the antenna, at the L1/E1 (**a**) and L5/E5 (**b**) frequency bands.

The difficulties to power the instrument complicated the measurements of the back part of the radiation patterns. The back lobes were measured only pointing to the boresight, and are shown in Figure 23.

**Figure 24:**
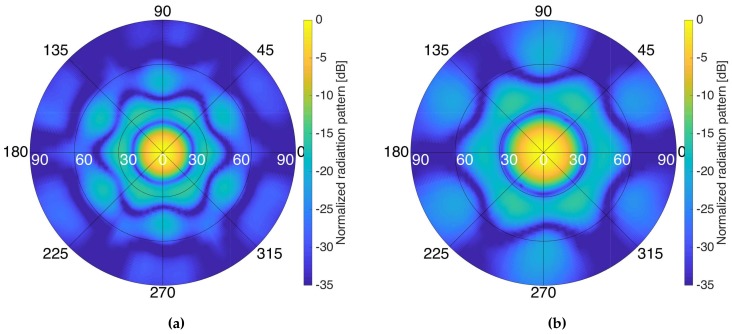
Frontal hemisphere of the measured radiation pattern of the down-looking array, at the L1/E1 (**a**) and at L5/E5 (**b**) frequency bands.

It was therefore complicated to accurately estimate the directivity of the array since only the frontal hemisphere of the radiattion pattern was measured (Figure 24). The estimated directivities for both arrays while pointing to the boresight can be seen in Table 2.

**Figure 25:**
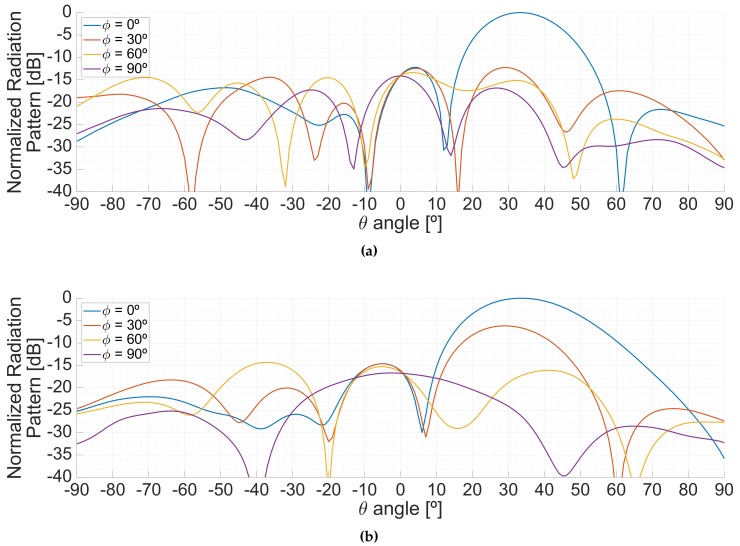
Side lobes for different azimuthal cuts of the antenna pattern when pointing to an off-boresight angle of 35${}^{\circ }$ at the L1/E1 (**a**) and L5/E5 (**b**) frequency bands, respectively.

Figure 25 shows different antenna radiation pattern cuts when pointing to an off-boresight angle of 35${}^{\circ }$. The side lobe levels are lower than 12 dB and 14 dB at L1/E1 and L1/E1 frequency bands, respectively.

### 4.2. Outdoor Experiments

**Figure 26:**
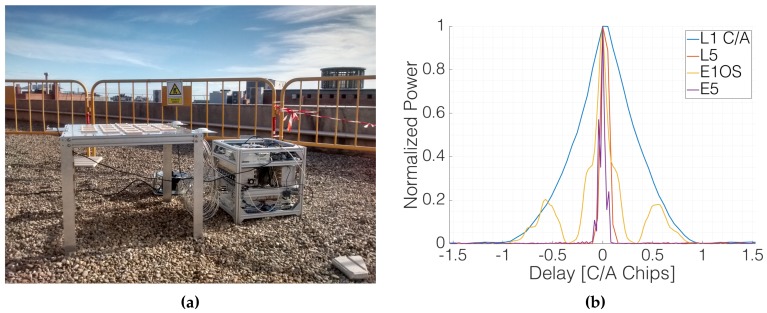
MIR up-looking array and rack during the rooftop experiments (**a**), and retrieved waveforms from GPS and Galileo satellites in both L1/E1 and L5/E5 frequency bands (**b**) during the rooftop experiments [23].

Several experiments were carried out in the rooftop of the laboratory to ensure the proper working of the instrument. The first one aimed to check that the instrument was able to retrieve data from all systems and frequency bands with enough power. To do so, the up-looking array was placed with a random orientation with respect to the North (Figure 26), pointing to GPS and Galileo satellites at both L1/E1 and at L5/E5 frequency bands. The received signals were sampled and stored at 16 bits and 20 Msps, and cross-correlated with clean replicas of the PRN codes. Figure 26 also shows four retrieved different waveforms for GPS and Galileo in both frequency bands. The correlation peaks are displayed in the time origin of a single figure to for easier intercomparison. The experiment also aimed at checking if extra gain was required to reach the sensitivity of the SDR and to take advantage of the whole dynamic range of the Analog to Digital Converters (ADC), which was not. In order to check if more gain was required for the down-looking RF chain, attenuators were placed before the SDRs in order to determine the sensitivity level. Since the reflected signals are expected to be 0–30 dB weaker than those collected by the up-looking RF chain, two extra RF amplifiers with a total gain of ≈35 dB were added just before the SDRs in the down-looking chains.

**Figure 27:**
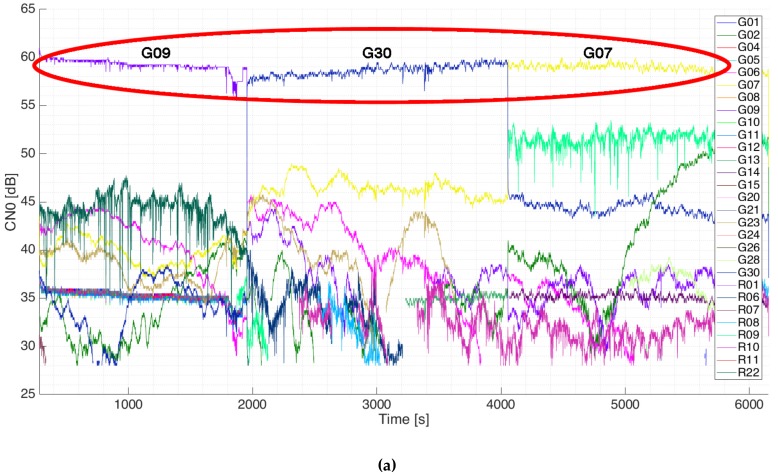

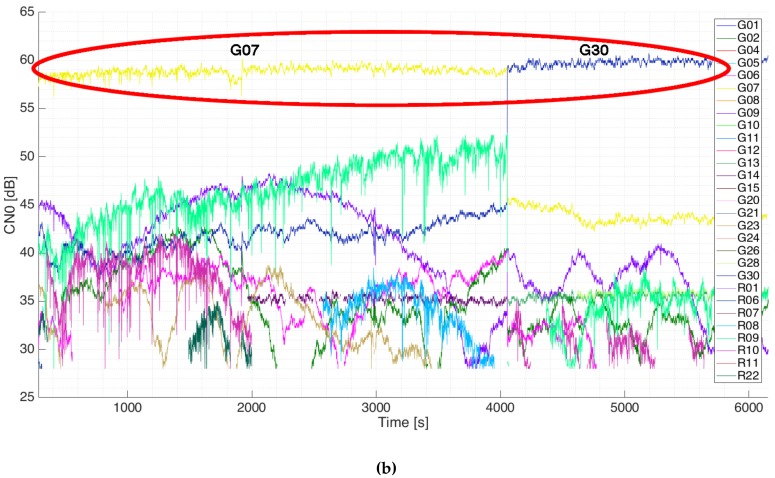
Tracked C/N0 in the L1-1 beam (**a**) and L1-2 beam (**b**), both red circled, during the experiment in the laboratory rooftop [24].

Later on, the satellite selection algorithm was tested by leaving the instrument tracking the optimum satellites and monitoring the C/N0 at the output of the beamformers. To do so, the outputs of the L1-1 and L1-2 beams were connected to a dual-antenna GNSS receiver capable of tracking GPS and GLONASS satellites (the selection of Galileo satellites was deactivated in order to be able to track the pointed satellites). The C/N0 of all satellites was monitored for a long time. Figure 27 shows the C/N0 tracked in the L1-1 beam (top) and the L1-2 beam (bottom). As it can be seen, the tracked satellite receives more power than the others. The satellite selection algorithm was set to chose the optimum satellites every 5 min. Two changes in the tracked satellites can be observed in the figure, where the C/N0 of the satellites drops or raises suddenly when the algorithm decided to track a different satellite.

## 5. Summary

This work has presented the MIR instrument, a new airborne GNSS-Reflectometer that has been built during the last years in Universitat Politècnica de Catalunya to emulate the behavior of the future PARIS-IoD [25], GNSS rEflectometry, Radio Occultation, and Scatterometry onboard the International Space Station (GEROS-ISS) [26], or GNSS Transpolar Earth Reflectometry exploriNg system (G-TERN) [27] missions thanks to its large directive antennas, multi-beam and dual-frequency (L1/E1 and L5/E5a) capabilities. Obtaining the “compensation parameters” eases the later periodical instrument calibration by injecting PRN signals during operations, which have been proved useful to calibrate the instrument.

The system’s analog beamformers use vector modulators to change the amplitude and phase of the received signals by the antennas. A 4-model parameter model is proposed to characterize the vector modulators. This model in conjunction with the proposed calibration procedure reduces the amplitude errors in the beamforming from 0.55 dB and 0.11 dB to 0.03 dB and 0.02 at L1/E1 and at L5/E5a, respectively. The phase errors are reduced in the beamforming from 13${}^{\circ }$ and 16${}^{\circ }$ to 4.9${}^{\circ }$ and 4.2${}^{\circ }$ at L1/E1 and at L5/E5a, respectively. The consequent loss of directivity in the array is therefore reduced from 0.28 dB and 0.34 dB to 0.03 dB and 0.02 dB at L1/E1 and at L5/E5a, respectively.

The array directivity pointing to the boresight reaches 20 dB and 18 dB at L1/E1 and at L5/E5a, respectively. The measured beamwidth pointing to the boresight is 17${}^{\circ }$ and 23${}^{\circ }$ at L1/E1 and at L5/E5a, respectively. When the beam is steered 35${}^{\circ }$ off-boresight, the beamwidth increases up to 20${}^{\circ }$ and 27${}^{\circ }$ at L1/E1 and at L5/E5a, respectively, and the side lobes are lower than 12 dB and 14 dB, respectively. The beamsteering capabilites have been demonstrated up to 50${}^{\circ }$ with a maximum directivity loss of 5 dB at L1, larger than the desired 35${}^{\circ }$. The automatic satellite tracking capabilities have also been proved.

Future work involves field experiments, development of data processing algorithms, implementation in the URSPs of these algorithms for real-time processing, and the implementation in the USRPs of the fourth step of the pre-calibration and the in-flight calibration to calibrate and compensate the component aging.

## Figures and Tables

**Table 1:** Measured directivity and estimated efficiency of the antenna elements.

Frequency Band	Directivity	Efficiency
L5/E5a	7.8 dB	80%
E5	8 dB	70%
L1/E1	7 dB	65%

**Table 2:** Measured directivity of the arrays.

Frequency Band	Up-Looking Array	Down-Looking Array
L1/E1	20.3 dB	20.4 dB
L5/E5b	18.2 dB	18.1 dB

**Table 3:** Beamformer inaccuracy impact in the array directivity before and after calibration.

	Uncalibrated				Calibrated			
	**Beamformer Error**		**Array Directivity Loss**		**Beamformer Error**		**Array Directivity Loss**	
	**L1/E1**	**L5/E5**	**L1/E1**	**L5/E5**	**L1/E1**	**L5/E5**	**L1/E1**	**L5/E5**
Amplitude	0.55 dB	0.11 dB	0.061 dB	0.0027 dB	0.02 dB	0.03 dB	<0.001 dB	<0.001 dB
Phase	13${}^{\circ }$	16${}^{\circ }$	0.22 dB	0.34 dB	4.9${}^{\circ }$	4.2${}^{\circ }$	0.03 dB	0.02 dB

## Abbreviations

The following abbreviations are used in this manuscript:

ADC	Analog-to-Digital Converter
BPSK	Binary Phase Shifting Keying
$B_{w}$	−3 dB Bandwidth
cGNSS-R	conventional Global Navigation Satellite Systems—Reflectometry
COTS	Commercial Off-The-Shelf
DAC	Digital-to-Analog Converter
DCM	Direction Cosine Matrix
DUT	Device Under Test
ECEF	Earth Centered Earth Fixed
$f_{c}$	Cut-off frequency at −3 dB
$f_{LO}$	LO frequency
FM	Frequency Modulation
G	Gain
GEROSS-ISS	GNSS rEflectometry, Radio Occultation and Scatterometry onboard the International Space Station
GLONASS	GLObalnaya NAvigatsionnaya Sputnikovaya Sistema
GNSS	Global Navigation Satellite Systems
GNSS-R	Global Navigation Satellite Systems—Reflectometry
GPS	Global Positioning System
GPS-DO	GPS Disciplined Oscillator
GSM	Global System for Mobile communications
G-TERN	GNSS Transpolar Earth Reflectometry exploriNg system
I	In-phase component
IF	Intermediate Frequency
iGNSS-R	interferometric Global Navigation Satellite Systems—Reflectometry
IMU	Inertial Measurement Unit
L	Losses, attenuation
LHCP	Left Hand Circular Polarized
LNA	Low Noise Amplifier
LO	Local Oscillator
MIR	Microwave Interferometric Reflectometer
Msps	Mega Samples Per Second
NED	North East Down
NF	Noise Figure
PARIS-IoD	PAssive Reflectometry and Interferometry System In-orbit Demonstration
PPS	Pulse Per Second
PRN	PseudoRandom Noise
Q	Quadrature phase component
RF	Radio Frequency
RFI	Radio Frequency Interference
RMS	Root Mean Square
RHCP	Right Hand Circular Polarized
SAW	Surface Acoustic Wave
SBAS	Satellite-Based Augmentation Systems
SDR	Software Defined Radio
SNR	Signal-to-Noise Ratio
$T_{c}$	Chip time of a PRN code
$T_{coh}$	Coherent integration time
$T_{code}$	PRN time length
USRP	Universal Software Radio Peripheral
VNA	Vector Network Analyzer
$\lambda_{L1}$	Wavelength of GPS at L1 central frequency (19.04 cm)
$\lambda_{L5}$	Wavelength of GPS at L5 central frequency (25.5 cm)

## Appendix A. Formulas Used in the Derivation of the Cross-Correlation Function for the Calibration Process

The cross-correlation function of the sampled calibration signal is

(A1) $$\begin{array}{cc} R_{yx}\left(t,\tau ,\Delta f\right) & =\left(\left(x_{T}\left(t\right)*h^{\prime }\left(t\right)\right)\cdot e^{-j\left(2\pi \Delta ft+\Delta \phi \right)}+n^{\prime }\left(t\right)\right)*\left(x^{*}\left(-t\right)\right)=\left\{\mathrm{using}\;\mathrm{Equation}\;(\mathrm{A2})\right\}\\ & =\left(h^{\prime }\left(t\right)*x_{T}\left(t\right)*\left(x^{*}\left(-t\right)\cdot e^{j2\pi \Delta ft}\right)\right)\cdot e^{-j(2\pi \Delta ft+\Delta \phi )}+n^{\prime }\left(t\right)*x^{*}\left(-t\right)=\left\{\mathrm{using}\;\mathrm{Equation}\;(\mathrm{A4})\right\}\\ & =\left(h^{\prime }\left(t\right)*\left(\sum Y\left(t-nT_{code},\Delta f\right)\cdot e^{j2\pi \Delta fnT_{code}}\right)\cdot e^{j2\pi \Delta ft}\right)\cdot e^{-j(2\pi \Delta ft+\Delta \phi )}+n^{\prime }\left(t\right)*x_{c}^{*}\left(-t\right)\\ & =\left(\left(h^{\prime }\left(t\right)\cdot e^{-j2\pi \Delta ft}\right)\overset{t}{*}\left(\sum Y\left(t-nT_{code},\Delta f\right)\cdot e^{j2\pi \Delta fnT_{code}}\right)\right)\left(\tau ,\Delta f\right)\cdot e^{-j\Delta \phi }+R_{n^{\prime }x}\left(t,\tau \right), \end{array}$$

(A2) $$\begin{array}{cc} x\left(t\right)*\left(h\left(t\right)\cdot e^{-j2\pi ft}\right) & =\left(x\left(t\right)\cdot e^{-j2\pi ft}\cdot e^{j2\pi ft}\right)*\left(h\left(t\right)\cdot e^{-j2\pi ft}\right)=\left\{\mathrm{using}\;\mathrm{Equation}\;(\mathrm{A3})\right\}\\ & =\left(\left(x\left(t\right)\cdot e^{j2\pi ft}\right)*(h\left(t\right)\right)\cdot e^{-j2\pi ft}, \end{array}$$

(A3) $$\begin{array}{cc} \left(x\left(t\right)\cdot e^{-j2\pi ft}\right)*\left(h\left(t\right)\cdot e^{-j2\pi ft}\right) & =\int x\left(t^{\prime }\right)\cdot e^{-j2\pi ft^{\prime }}\cdot h\left(t-t^{\prime }\right)\cdot e^{-j2\pi f(t-t^{\prime })}dt^{\prime }\\ & =\int x\left(t^{\prime }\right)\cdot e^{-j2\pi ft^{\prime }}\cdot h\left(t-t^{\prime }\right)\cdot e^{-j2\pi ft}\cdot e^{j2\pi ft^{\prime }}dt^{\prime }\\ & =e^{-j2\pi ft}\cdot \int x\left(t^{\prime }\right)\cdot h\left(t-t^{\prime }\right)dt^{\prime }=\left(x(t)*h(t)\right)\cdot e^{-j2\pi ft}, \end{array}$$

(A4) $$\begin{array}{cc} x_{T}\left(t\right)*\left(x^{*}\left(-t\right)\cdot e^{j2\pi \Delta ft}\right) & =\sum \delta \left(t-nT_{code}\right)*\left(x\left(t\right)*\left(x^{*}\left(-t\right)\cdot e^{j2\pi \Delta ft}\right)\right)\\ & =\sum \delta \left(t-nT_{code}\right)*\int_{0}^{T_{coh}}x\left(t^{\prime }\right)\cdot x\left(t^{\prime }-t\right)\cdot e^{j2\pi \Delta f(t-t^{\prime })}dt^{\prime }\\ & =\sum \delta \left(t-nT_{code}\right)*\left(e^{j2\pi \Delta ft}\cdot \int_{0}^{T_{coh}}x\left(t^{\prime }\right)\cdot x\left(t^{\prime }-t\right)\cdot e^{-j2\pi \Delta ft^{\prime }}dt^{\prime }\right)\\ & =\sum \delta \left(t-nT_{code}\right)\overset{t}{*}\left(Y\left(t,\Delta f\right)\cdot e^{j2\pi \Delta ft}\right)\\ & =\sum Y\left(t-nT_{code},\Delta f\right)\cdot e^{j2\pi \Delta f(t-nT_{code})}\\ & =\left(\sum Y\left(t-nT_{code},\Delta f\right)\cdot e^{j2\pi \Delta fnT_{code}}\right)\cdot e^{j2\pi \Delta ft}. \end{array}$$

If it is assumed a simplified model of the channel $h^{\prime }\left(t\right)=A_{c}\cdot \delta \left(t-\tau_{c}\right)\cdot e^{j\phi_{c}}$, and the model for the WAF defined in Equation (30), the cross-correlation of the calibration signal becomes

(A5) $$\begin{array}{cc} R_{yx}\left(t,\tau ,\Delta f\right) & ={\left(\left(A_{c}\cdot \delta \left(t-\tau_{c}\right)\cdot e^{j\Phi_{c}}\cdot e^{-j2\pi \Delta ft}\right)\overset{t}{*}\left(\sum Y\left(t-nT_{code},\Delta f\right)\cdot e^{j2\pi \Delta fnT_{code}}\right)\right)}_{t=\tau }\cdot e^{-j\Delta \phi }+R_{w^{\prime }x}\left(t,\tau \right)\\ & ={\left(\left(A_{c}\cdot \delta \left(t-\tau_{c}\right)\cdot e^{-j(2\pi \Delta f\tau_{c}+\Phi_{c})}\right)\overset{t}{*}\left(\sum Y\left(t-nT_{code},\Delta f\right)\cdot e^{j2\pi \Delta fnT_{code}}\right)\right)}_{t=\tau }\cdot e^{-j\Delta \phi }+R_{w^{\prime }x}\left(t,\tau \right)\\ & =A_{c}\cdot \left(\sum Y\left(\tau -nT_{code}-\tau_{c},\Delta f\right)\cdot e^{j2\pi \Delta fnT_{code}}\right)\cdot e^{-j\Delta \phi }\cdot e^{-j(2\pi \Delta f\tau_{c}+\Phi_{c})}+R_{w^{\prime }x}\left(t,\tau \right)\\ & =A_{c}\cdot \left(\sum ▵\left(\frac{\tau -\tau_{c}-nT_{code}}{T_{c}}\right)\cdot sinc\left(\Delta f\cdot T_{c}\right)\cdot e^{j2\pi \Delta fnT_{code}}\right)\cdot e^{-j(2\pi \Delta f\tau_{c}+\Phi_{c}+\Delta \phi )}+R_{w^{\prime }x}\left(t,\tau \right). \end{array}$$

## References

[1] Hall C., Cordey R. Multistatic Scatterometry. Proceedings of the International Geoscience and Remote Sensing Symposium: Remote Sensing: Moving Toward the 21st Century.

[2] Martin-Neira M. (1993). A Passive Reflectometry and Interferometry System (PARIS): Application to Ocean Altimetry. ESA J..

[3] Onrubia R., Querol J., Pascual D., Alonso-Arroyo A., Park H., Camps A. (2016). DME/TACAN Impact Analysis on GNSS Reflectometry. IEEE J. Sel. Top. Appl. Earth Obs. Remote Sens..

[4] Pascual D., Park H., Onrubia R., Arroyo A.A., Querol J., Camps A. (2016). Crosstalk Statistics and Impact in Interferometric GNSS-R. IEEE J. Sel. Top. Appl. Earth Obs. Remote Sens..

[5] Katzberg S., Garrison J. (1996). Utilizing GPS To Determine Ionospheric Delay over the Ocean.

[6] European Union (2014). European GNSS (Galileo) Open Service Signal-In-Space Interface Control Document (OS SIS ICD).

[7] Pascual D., Camps A., Martin F., Park H., Arroyo A.A.A., Onrubia R. (2014). Precision Bounds in GNSS-R Ocean Altimetry. IEEE J. Sel. Top. Appl. Earth Obs. Remote Sens..

[8] Onrubia R., Camps A. (2015). Antena Multibanda Tipo Parche con Sistema de Alimentación Cruzada. Spain Patent.

[9] Alonso-Arroyo A., Camps A., Monerris A., Rudiger C., Walker J.P., Onrubia R., Querol J., Park H., Pascual D. (2016). On the Correlation Between GNSS-R Reflectivity and L-Band Microwave Radiometry. IEEE J. Sel. Top. Appl. Earth Obs. Remote Sens..

[10] Analog Devices ADL5390 Rev. A. http://www.analog.com/media/en/technical-documentation/data-sheets/ADL5390.pdf.

[11] Wandboard Quad (I.MX6). https://www.wandboard.org/products/wandboard/WB-IMX6Q-BW/.

[12] Trimble BD982. https://www.trimble.com/gnss-inertial/bd982.aspx.

[13] Onrubia R., Garrucho L., Pascual D., Park H., Querol J., Alonso-Arroyo A., Camps A. Advances in the MIR instrument: Integration, control subsystem and analysis of the flight dynamics for beamsteering purposes. Proceedings of the 2015 IEEE International Geoscience and Remote Sensing Symposium (IGARSS).

[14] GPS (2013). NAVSTAR GPS Control Segment to User Support Community Interfaces.

[15] Seika NG-4. http://www.seika.de/english/htmle/NGe.htm.

[16] 9 Degrees of Freedom—Razor IMU. https://www.sparkfun.com/products/retired/10736.

[17] Ettus Research USRP X310. https://www.ettus.com/content/files/X300_X310_Spec_Sheet.pdf.

[18] Ettus Research Octoclock. https://www.ettus.com/content/files/Octoclock_Spec_Sheet.pdf.

[19] Proakis J.G., Manolakis D.G. (1996). Digital Signal Processing Principles, Algorithms and Applications.

[20] Zavorotny V.U., Gleason S., Cardellach E., Camps A. (2014). Tutorial on remote sensing using GNSS bistatic radar of opportunity. IEEE Geosci. Remote Sens. Mag..

[21] Querol J. (2018). Radio Frequency Interference Detection and Mitigation Techniques for Navigation and Earth Observation. Ph.D. Thesis.

[22] Camps A., Park H., Valencia i Domenech E., Pascual D., Martin F., Rius A., Ribo S., Benito J., Andres-Beivide A., Saameno P. (2014). Optimization and Performance Analysis of Interferometric GNSS-R Altimeters: Application to the PARIS IoD Mission. IEEE J. Sel. Top. Appl. Earth Obs. Remote Sens..

[23] Onrubia R., Pascual D., Querol J., Park H., Camps A., Rudiger C., Walker J. Preliminary End-to-End Results of the MIR Instrument: the Microwave Interferometric Reflectometer. Proceedings of the IGARSS 2018—2018 IEEE International Geoscience and Remote Sensing Symposium.

[24] Onrubia R., Pascual D., Querol J., Park H., Camps A. Beamformer characterization of the MIR instrument: The microwave interferometric reflectometer. Proceedings of the 2017 IEEE International Geoscience and Remote Sensing Symposium (IGARSS).

[25] Martin-Neira M., D’Addio S., Buck C., Floury N., Prieto-Cerdeira R. (2011). The PARIS Ocean Altimeter In-Orbit Demonstrator. IEEE Trans. Geosci. Remote Sens..

[26] Wickert J., Andersen O., Beyerle G., Chapron B., Cardellach E., D’Addio S., Foerste C., Gommenginger C., Gruber T., Helm A. (2013). GEROS-ISS: Innovative GNSS Reflectometry/Occultation Payload Onboard the International Space Station for the Global Geodetic Observing System.

[27] Cardellach E., Wickert J., Baggen R., Benito J., Camps A., Catarino N., Chapron B., Dielacher A., Fabra F., Flato G. (2018). GNSS Transpolar Earth Reflectometry exploriNg System (G-TERN): Mission Concept. IEEE Access.

